# Recent Progress in Cellulose-Based Aerogels for Sustainable Oil–Water Separation Technologies

**DOI:** 10.3390/polym17202723

**Published:** 2025-10-10

**Authors:** Karvembu Palanisamy, Gowthami Palanisamy, Yeong Min Im, Sadhasivam Thangarasu, Urmila Gupta Phutela, Tae Hwan Oh

**Affiliations:** 1 Department of Microbiology, Punjab Agricultural University, Ludhiana 141004, India; vembubiomicro@gmail.com; 2 Department of Chemical Engineering, Yeungnam University, Gyeongsan 8541, Republic of Korea; yym2150@naver.com (Y.M.I.); sadhasivam.nano@gmail.com (S.T.)

**Keywords:** polymers, bio-polymers, polymer aerogels, hybrid composite, oil–water separation, environmental remediation, surface-modification, synthetic polymers

## Abstract

Polymer-based aerogels have recently received considerable research attention as a favorable option for oil–water separation due to their enhanced porous 3D structure with great specific surface area, low density and outstanding sorption behavior. Additionally, polymer-containing aerogels exhibit more favorable characteristic properties, such as being lipophilic–hydrophobic (superhydrophobic–superoleophilic), hydrophilic–lipophobic (superhydrophilic–underwater oleophobic), or other specific wetness forms, including anisotropic and dual-wettability. In this review, cellulose and cellulose-based materials used as an aerogel for oil–water separation are comprehensively reviewed. This review highlights the significance of cellulose and cellulose-based combinations through structure–property interactions, surface modifications (using different hydrophilic and hydrophobic agents), and aerogel formation, focusing on the light density and high surface area of aerogels for effective oil–water separation. This article provides an in-depth review of four primary classifications of cellulose-based aerogels, namely, cellulose aerogels (regenerated cellulose and bacterial cellulose), cellulose with biopolymer-based aerogels (chitosan, lignin, and alginate), cellulose with synthetic polymer aerogels (polyvinyl alcohol, polyetherimide, polydopamine and others), and cellulose with organic/inorganic (such as SiO_2_, MTMS, and tannic acid) material-based aerogels. Furthermore, the aspects of performance, scalability, and durability have been explained, alongside potential prospect directions for the advancement of cellulose aerogels aimed at their widespread application. This review article stands apart from previously published review works and represents the comprehensive review on cellulose-based aerogels for oil–water separation, featuring wide-ranging classifications.

## 1. Introduction

**Figure 1:**
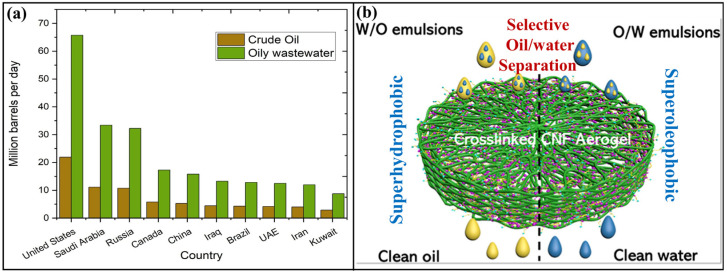
(**a**) Oily wastewater as a fraction of 10 nations oil production (Administration, U.S.E.I. U.S. Energy Information Administration, International Energy Statistics, 2024). Reprinted with permission from ref. [15]. Copyright © 2025 The Authors. Published by Elsevier Ltd. (**b**) Schematic of cellulose-based aerogels on oil- and water-based separation processes. Reprinted with permission from ref. [38]. Copyright © 2024, American Chemical Society. (License number: 6086980873291).

The rapid expansion of industrialization, an accelerating growth of population, and the intensification of agricultural practices all contribute to the widespread growth of air, soil, and water pollution, which is emerging as a significant influence on the environment on a worldwide scale [1,2]. The pollution of water presents a significant risk, including to the environment, ecology, and human health [3,4]. Additionally, the aquatic systems have been polluted by a wide variety of pollutants, which include chemical compounds, biological materials, and physical substances [5]. Over the last several years, oily wastewater has emerged as a significant reason for concern in the area of wastewater treatment systems [6,7,8,9]. Oily wastewater (oil-in-water and water-in-oil emulsions) generated by food industries, petroleum companies, and leaks during oil transportation represents a substantial contributor to a pressing environmental challenge [10,11,12,13]. On a global scale, the amount of oily wastewater has significantly expanded as a result of the increasing extraction and processing of oil [14]. For every barrel of oil product, there is an estimated three barrels of oily wastewater, as shown in Figure 1a [15,16,17] [Administration, U.S.E.I. U.S. Energy Information Administration, International Energy Statistics, 2024] [Summary of Input on Oil and Gas Extraction Wastewater Management Practices Under the Clean Water Act, in EPA-821-S19–001 (2020) Washington, D.C. 20460]. Roughly 90 million barrels per day of oily effluent is estimated to be produced worldwide by offshore rigs [15,18]. Moreover, offshore sites discharge more than 44 million barrels of oily effluent per day [15,19,20]. It is anticipated that this tendency will continue, given the amount of oily wastewater produced by the oil industry has significantly grown. The presence of oily wastewater adversely affects aquatic ecosystems by restricting oxygen transmission, causing damage to biodiversity, and destabilizing the ecological balance [21,22,23,24]. The methods for oil/water separation can be considered to fall into the following three main categories: chemical, physical, and biological approaches [25]. According to the process, it has been further classified in many ways [6,26,27]. However, many techniques are associated with certain drawbacks, such as prolonged processing time, consuming more energy, recyclability, and/or environmental compatibility [28,29]. The challenges presented necessitate the urgent advancement of innovative, sustainable, and cost-effective materials and methodologies for oil/water separation, aiming to achieve high-performance, eco-friendly solutions suitable for practical applications. In this connection, there are a variety of physical or mechanical procedures used in the process of oil–water separation. Some examples of these techniques include centrifugation, gravity separation, flotation, filtration, and ultrasonic separation [30,31,32]. The separation of oil and water by the use of filtration through porous materials such as meshes, membranes and aerogels is more effective and feasible [33,34,35,36,37]. For oil–water separation, membrane materials effectively address the issues of low efficiency and high energy consumption associated with traditional separation techniques like gravity sedimentation and centrifugal separation. However, membrane based technologies p encountering major limitations due to material properties, separation conditions, fouling and application conditions. These shortcomings hinder consistent performance, long-term stability, and scalability. However, aerogel-based oil–water separation is more advantageous due to its high absorption capacity, low energy requirements, recyclability, and adaptability. The possible mechanism for oil–water separation via porous media is illustrated in Figure 1b [38]. These materials are ideal for a variety of uses, including treatment of wastewater and oil spill cleaning, due to their adaptability, high effectiveness, and low energy costs [39,40].

### 1.1. Aerogels for Oil–Water Separation

Among the several alternatives, aerogels have garnered a significant amount of interest in recent times for the process of oil–water separation. Aerogels are 3D materials, which are exceptionally porous, ultralight, and created by replacing the liquid phase of a gel with air [41,42,43]. This results in solids providing an extraordinary surface area, low density, great mass transfer capabilities, and an interconnected pore network [44]. In aerogel, the mechanism involves physical adsorption, chemical adsorption, and electrostatic interactions that can be further influenced by larger aerogel surface area, pore structure, and functional modification of aerogel. Various factors such as adsorbate type, concentration, temperature, aerogel composition, and functional groups also influence the sorption mechanism. The surface wettability of aerogels used for oil–water separation is the primary factor that determines the classification of these aerogels. These aerogels might be lipophilic–hydrophobic (superhydrophobic–superoleophilic) aerogels, hydrophilic–lipophobic (superhydrophilic–underwater oleophobic) aerogels, or other particular wetness forms of aerogels such as anisotropic and dual-wettability aerogels [45,46]. The distinct advantageous properties, combined with adjustable surface chemistry, lead to the effective use of aerogels in environmental applications, especially in the separation of oil and water [46,47]. Aerogels are responsible for the quick and high-capacity sorption/separation of oil pollutants. On the basis of aerogels enhanced porous structure and greater surface area, aerogels are accountable for efficient function. Additionally, their flotation and ease of recovery from water surfaces are confirmed by their very low density. Depending on the separation principle, aerogels can function as underwater superoleophobic, hydrophilic-lipophilic, or superhydrophobic/superoleophilic. These features demonstrate effective separation and selective oil capture, even in stable oil-in-water emulsions [45]. In addition, aerogels often displayed great mechanical and chemical stability, which enabled them to be reused several times with a minimal decrease in performance.

For the purpose of oil–water separation, many forms of aerogels including organic or polymer-based aerogels, (like polyvinyl alcohol (PVA), polylactic acid (PLA), and cellulose), inorganic-based aerogels (like silica (SiO_2_) and metal oxides), and carbon-based aerogels (like graphene or carbon nanotubes), have been used and exhibited with efficient performances [48,49,50]. Despite their impressive sorption capabilities, conventional aerogels encounter specific challenges, especially when it comes to large-scale practical applications. The difficulties include higher costs and limited ability for biodegradation. Aerogels made of bio-based materials have recently attracted attention as a potential solution because of their eco-friendliness, biodegradability, and compatibility with living organisms [51,52,53,54]. Cellulose, proteins, starch, alginate, and chitosan are examples of common precursors for bio-aerogels. These precursors help in the creation of environmentally acceptable remediation materials for developing the bio-based aerogels and decrease dependency on synthetic polymers derived from petroleum [47,51,55,56]. In the area of oil/water separation, the third-generation aerogels, such as cellulose-based aerogel, exhibited numerous advantages over other bio-aerogels, such as starch, alginate, and chitosan. This was attributed by the versatile mechanical stability of cellulose with 5.2 kPa to 16.67 kPa of compressive strength [57], while starch, alginate, and chitosan are weak and brittle in their pure form. In order to improve the mechanical stability, modifications such as cross-linking/reinforcement with cellulose (microcrystalline or nanofibrils) have been performed in starch, alginate, and chitosan aerogels. Cellulose aerogels exhibit improved stability in a moist condition owing to their hierarchical fibrillary architecture. In the absence of modification, other biopolymer aerogels frequently develop swelling and a degradation of their structural integrity. Furthermore, the crystallinity and orientation of the cellulose chain contributed to the higher thermal stability (275 to 330°) [58] than other biopolymer aerogels. Moreover, the cellulose aerogels are highly tunable due to the presence of numerous, easily available –OH groups across the polymer chain and hierarchical fiber structure [59]. Furthermore, cellulose has been acting as a reinforcing agent for other biopolymer aerogels, such as those made from starch, alginate, and chitosan.

### 1.2. Importance of Cellulose-Based Aerogels

Among the many biopolymers, cellulose provides a significant number of advantages [57,60] pertaining to the absorption/separation of moisture, water, and oil. The numerous advantages of cellulose-based aerogels include their versatility, biodegradability, high mechanical strength, low weight, and varied surface chemistry, which allows for chemical modifications to enhance hydrophobicity and hydrophilicity [43]. Thus, the cellulose-based aerogels are well-suited for oil–water separation, where surface functionalization enables selective absorption of oil or water by tuning their hydrophobicity and hydrophilicity. The specific benefits of cellulose materials are high porosity, surface area, and tunable properties (cellulose source, cross-linking agents, and surface treatments), superhydrophobic (greatly water repellent) and underwater oleophobic (repellent to oil) behaviors [61,62,63,64,65]. Cellulose can effectively perform in various mechanisms, including selective absorption, gravity-driven separation, and de-emulsification [61]. The high-performance cellulose-based aerogels can be effectively prepared through cross-linking with different polymer materials, surface modification using silylation agents (such as methyltrimethoxysilane and trimethylchlorosilane), and composite formation with inorganic materials [61,62,66]. Cellulose-based aerogels present significant potential for the remediation of oil spills in aquatic environments, eliminating oil from industrial wastewater and separating oil and water in diverse industrial applications [62,63,65,66,67]. Bio-aerogels are fabricated through the primary sol–gel method. It involves the following three steps: cellulose dispersion into homogenous solution (sol), conversion of liquid into gel (gel) through the sol–gel method, and the drying process [68]. The most significant part of gel formation is gelation, in which the sol–gel transition occurred and resulted in a 3D porous network of interconnected structures through intermolecular or intramolecular hydrogen bonding. Generally, chemical additives such as N,N′-methylenebisacrylamide (MBA) and epichlorohydrin (ECH) have been added to liquid sol or the physical parameters (such as temperature, pH, cellulose concentration and dispersion, etc.) have been altered to form a homogenous colloid [69]. Later, the three-dimensional interconnected porous network was developed by polymerization and condensation of cellulose [68]. As a result, the as-developed solid gel can be named as alcogel, hydrogel, or acetogel based on the type of solvent utilized (alcohol, water, or acetone) during gelation. The gel developed through chemical cross-linking agents exhibited higher gel formation speed along with enhanced gel stability [57]. Moreover, the factors such as cellulose concentration, gel aging temperature, and time are responsible for the characteristics of the developed gel in sol–gel transition steps. Here, the aging process has been performed to stabilize and strengthen the interconnected gel network through different methods of cross-linking agents. Finally, a special drying process is required for removing the liquid present in the pores and replacing it with air without destroying its structure for developing aerogel. The aerogel structural morphology, such as pore size, specific surface area, pore density, pore volume, and porosity, strongly relies on this drying process. This involves different techniques such as atmospheric drying, freeze-drying, and supercritical drying [70,71,72,73,74].

Recent review have concentrated on biopolymers specifically cellulose based aerogels, which are exploring the (i) polysaccharide-based composite aerogels [47], which is explaining the preparation of polysaccharide-based aerogel materials for oil–water separation, (ii) removing water pollutants such as oils, pharmaceutical waste, organic dyes, and heavy metals via cellulose-based materials [75], (iii) theoretical basis of plant cellulose-based aerogel materials for oil–water mixture separation [76], (iv) hydrophobization of nanocellulose-based aerogels for oil spill applications [77], (v) superhydrophobic of nanocellulose aerogels for oil–water separation [78], and (vi) superwetting aerogel-based materials (such as carbon-, inorganic-, polymer-, and biomass-based aerogels) for oil–water separation [46]. Nevertheless, there are limited reviews on cellulose-based aerogels, and comprehensive explanations (both the concepts of hydrophilic-lipophobic (superhydrophilic–underwater oleophobic) aerogel and lipophilic–hydrophobic (superhydrophobic–superoleophilic) aerogel, which effectively categorize this subject, are even rarer. In this review, we summarize the application of cellulose-based aerogels in the oil–water separation process via lipophilic–hydrophobic and hydrophilic–lipophobic interactions in recent years. The literature review was carefully collected from developments over the past five years. A comprehensive review of the existing literature was conducted by using various types of cellulose and cellulose-based combinations (with synthetic polymer, biopolymer inorganic, and organic compounds) as high-performance aerogel materials for oil–water separation. Furthermore, this review categorized the primary subject into sub-sections, including cellulose aerogels (regenerated cellulose and bacterial cellulose), cellulose with biopolymer-based aerogels (chitosan, lignin, and alginate), cellulose with synthetic polymer aerogels (polyvinyl alcohol, polyetherimide, polydopamine, and others), and cellulose with organic/inorganic (such as SiO_2_, MTMS, and tannic acid) material-based aerogels. This review aims to provide an in-depth understanding of the techniques employed in the synthesis of these materials, types of cellulose-based aerogels, the efficacy of the oil–water separation, and the potential underlying processes involved.

## 2. Cellulose-Based Aerogels

Cellulose-based aerogels demonstrated numerous advantages owing to their renewability, biodegradability, high mechanical stability, low density, and rich surface chemistry, which allows for chemical modification to improve hydrophobicity [47,67]. Amphiphilic characteristics allow raw celluloses to absorb water and oil without surface modification [79]. To enhance selectivity for oil and oil absorption, significant attention has been directed towards modifying amphiphilic cellulose into highly hydrophobic materials through alterations in surface properties [80]. Hydrophobic groups with low surface energy are often added to the cellulose aerogel matrix by either chemical or physical methods [57]. The high abundance of hydroxyl groups in cellulose significantly enhances the surface modification of cellulose-based aerogels through diverse chemical routes such as silylation, oxidation, sulfonation, esterification, etc. [76]. In order to enhance the hydrophobicity of the cellulose-based aerogels, the –OH groups can be replaced with non-polar groups (water repelling groups) in order to enhance the oil–water separation performances. An efficient method such as chemical vapor deposition (CVD) is used to perform uniform hydrophobic coating of aerogel surface without affecting aerogel pore structure [81]. Here, CVD utilizes different coupling agents such as methyltrichlorosilane (MTCS), methyltrimethoxysilane (MTMS), n-Dodecyltriethoxysilane, trimethylchlorosilane (TMCS), and octadecyltrimethoxysilane (OTMS). Additionally, different techniques such as atomic layer deposition (ALD) and cold plasma treatment can be used for developing hydrophobicity. Moreover, hydrophobic modification can also be performed through various cross-linking agents such as isocyanate and 3,4-butanetetracarboxylic acid (BTCA) during gel formation [82]. Moreover, other hydrophobic techniques such as esterification and alkyl ketene dimer modification are also performed for attaining hydrophobicity in cellulose aerogel [83,84]. Compared to synthetic polymer-based or inorganic aerogels, these alteration strategies are relatively facile and less chemically rigorous, thereby offering a more sustainable and versatile platform for functional material design. Furthermore, cellulose-based aerogels showed superior mechanical flexibility compared to brittle inorganic counterparts. The easy functionalization, sustainable source, and strong mechanical properties make cellulose aerogels a promising category of materials for effective oil/water separation. Through a constant oil–water separation cycle, structural degradation and reduction in sorption ability can possibly occur in cellulose aerogels. Cellulose aerogels serve as effective, recyclable filters, which utilize their porous framework, modification of the surface, and durability to selectively separate water, including emulsions, from oil [62,85]. The overall functional properties of cellulose aerogel can be enhanced through various approaches such as reinforcing fibers, combination with other biopolymers, silane surface modification, or introducing the inorganic materials [86]. The alteration and combination can significantly enhance the performances of cellulose-based aerogels in terms of sorption capacity, selectivity, mechanical strength, structural stability, and tunable flexibility.

### 2.1. Regenerated Cellulose-Based Aerogels

**Figure 2:**
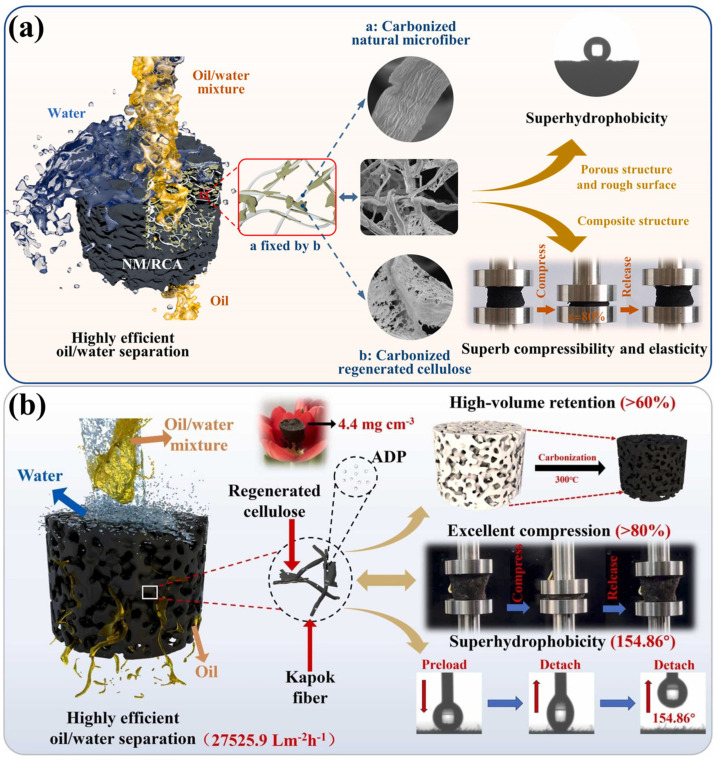
(**a**) Schematic representation of NM/RCA−based aerogel mechanism and advantageous (density of 8 to 10 mg cm^−3^, specific surface area of 768.89 m^2^ g^−1^, and WCA of 151.5°). Reprinted with permission from ref. [91]. Copyright © 2023 Elsevier B.V. (License number: 6086981089367). (**b**) Illustration of Kapok/regenerated cellulose-based carbon aerogel with a density of 4.4 mg cm^−3^, and a WCA of 154.9° for oil–water separation. Reprinted with permission from ref. [92]. Copyright © 2024 Elsevier B.V. (License number: 6086981337419).

Regenerated cellulose has been obtained by dissolving cellulose from wood pulp or cotton linters (natural cellulose sources) and re-precipitating it into solid form via coagulation, resulting in cellulose II type crystalline morphology [87]. The aerogels derived from regenerated cellulose demonstrated exceptional sorption capabilities for oils, which can be considered an as promising material for environmental remediation. The regeneration pathways for cellulose can process using different solvents such as N-methyl morpholine-N-oxide, ionic liquids, and deep eutectic solvents (DES) and its impacts [88,89,90]. Ma et al. [91] developed a novel 3D porous carbon aerogel comprising natural microfibrils (filiform)/regenerated cellulose (lamellate form) (NM/RCA) for use in oil/water separation. Figure 2a shows the overall benefits and mechanism of NM/RCA-based aerogel. The NM/RCA-based aerogel has been extracted by dissolving hardwood pulp using a co-solvent of N-methyl morpholine-N-oxide monohydrate (NMMO⋅H_2_O) and DES. The natural fibril surface roughness has been enhanced by DNS (DES and NMMO⋅H_2_O) in co-solvent used in dissolving hardwood pulp, which further increases the super hydrophobicity along with effective oil–water separation properties. After carbonization at 400 °C, it has been observed that the filiform and lamellate morphology and structure of the aerogel remain unchanged. This phenomenon provides an increase in compressibility, as well as excellent fatigue resistance, outstanding elasticity, and reusability. Furthermore, the developed carbon aerogel had an exceptionally low density of 8 to 10 mg/cm^3^. The aerogel has a remarkable porosity of 98.1% and a superior effective surface area of 768.89 m^2^/g. The unaltered shape and structure of carbonized aerogel demonstrated a super hydrophobic water contact angle of 151.5° and revealed a sorption capacity of between 63.1 and 133.2 g/g by sorbing different organic solvents and oils such as hexane, ethyl acetate, paraffin oil, phenoxin, hydraulic fluid, dichloromethane, dimethyl sulfoxide, pump oil, acetone, and edible oil at room temperature. Moreover, the NM/RCA aerogel showed enhanced flux of about 11,718.8 L/m^2^h during continuous oil/water separation [91]. In a related study, kapok fiber and regenerated cellulose (Figure 2b) have been applied to fabricate superhydrophobic cellulose-based carbon aerogel for oil/water separation [92]. The carbon aerogel has been prepared by carbonizing kapok and hardwood pulp at 300 °C (low temperature) using green cellulose solvent (N-methyl N-oxide monohydrate) and carbonization promoter (NH_4_H_2_PO_4_, (ADP)). The low-temperature carbonization attributes the remaining morphological and structural unchangedness of kapok and regenerated cellulose, which aids in outstanding mechanical properties through enhancing elasticity. Moreover, the ADP enhances the compressibility of aerogel, which generates oleophilic/superhydrophobic properties at reduced carbonization temperature. The ultralight porous carbon aerogel exhibits a sorption capacity ranging from 98.0 to 232.1 g^−1^ for different oils. Furthermore, it retains 87.3% of sorption capacity after 20 cycles [92]. Similarly, Ma et al. [93] developed eco-friendly, cost-effective carbon aerogel from kapok fibers and regenerated cellulose from hardwood pulp using DNS co-solvent. The as-developed aerogel exhibited outstanding elasticity, compressibility, and fatigue resistance. This characteristic behavior was attributed to the 3D porous network developed from the carbonization process. Moreover, the developed carbon aerogel exhibited a low density of 3.78 mg/cm^3^, 98% high porosity, and increased hydrophobicity of 144.7° with a remarkable oil sorption capacity of 137.5 to 317.7 g/g. This aerogel attained an increased flux rate of 21972.7 L/m^2^h (gravity driven) during n-hexane/water filtration [93].

### 2.2. Bacterial Cellulose-Based Aerogel

**Figure 3:**
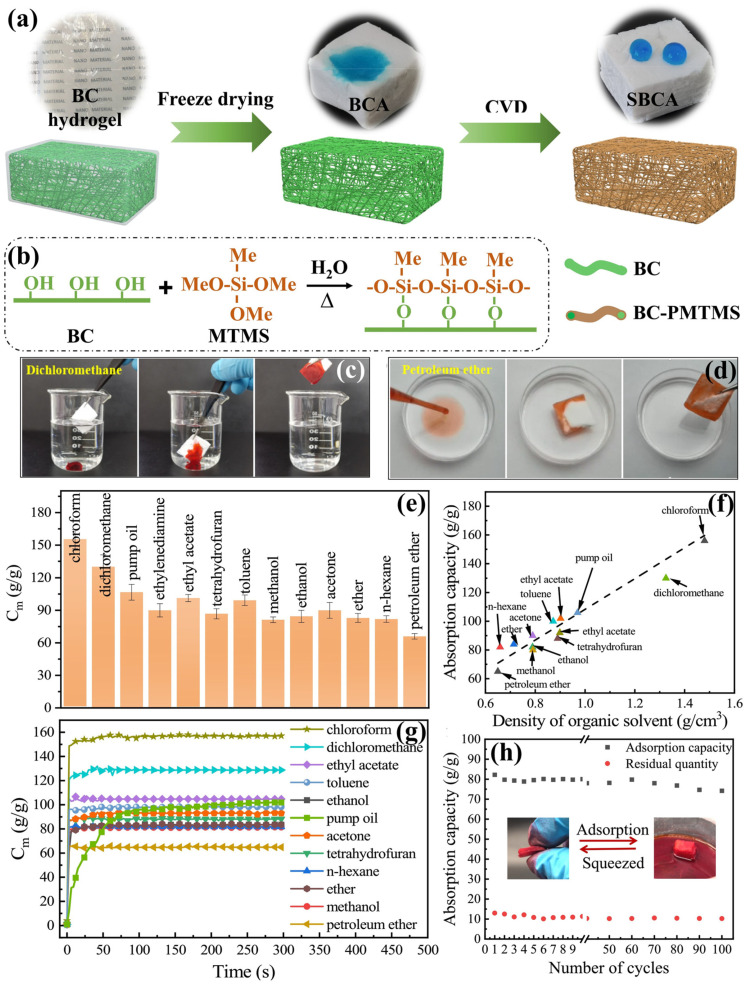
(**a**,**b**) Schematic of silylated bacterial cellulose aerogel (SBCA) preparation steps. SBCA oil absorption/separation performances: Removal of (**c**) dichloromethane and (**d**) petroleum ether in the water. (**e**) Absorption capabilities of SBCA for oils and organic solvents by mass. (**f**) Liquid density-dependent organic solvent sorption capabilities and (**g**) absorption kinetics. (**h**) n-hexane cyclic absorption capabilities of the SBCA. Reprinted with permission from ref. [96]. Copyright © 2024 Elsevier B.V. (License number: 6086990045390).

A 3D network of sustainable bacterial cellulose (BC) aerogels was developed via kombucha fermentation of fruit waste and applied for oil–water separation. The resulting aerogels were fabricated using freeze-drying techniques, followed by hydrophobic surface modification via chemical vapor deposition. The as-prepared aerogels exhibited an ultra-low density of 5.693 mg cm^−3^ and increased porosity ranging from 98 to 99%. Additionally, the modified aerogels showed enhanced hydrophobicity with a contact angle of ~146.4°. Remarkably, the aerogels demonstrated excellent oil sorption capacity, absorbing 30 to 177 times their own weight, corresponding to 36.5–118.3 g/g depending on the type of oil [94]. A dual cross-linking assembly technique has been developed to fabricate highly flexible and hydrophobic BC aerogels [95]. To enhance dimensional stability and mechanical durability, 1,2,3,4-butanetetracarboxylic acid (BTCA) is initially subjected to covalent esterification with BC hydroxyls, resulting in the formation of a robust network. Additionally, BTCA serves as an effective acid catalyst, enhancing the methyltrimethoxylsilane (MTMS) chemical vapor deposition (CVD) process and improving the elasticity of the aerogel. The BC/BTCA/MTMS aerogel demonstrates notable fatigue resistance, maintaining over 80% elasticity after 50 cycles. The hydrophobically modified aerogel (WCA 142°) exhibits exceptional oil absorbency. Furthermore, it retains over 87% and 81% of its initial sorption capacity for n-hexane and dichloroethane after 50 absorption–squeeze cycles. It is noteworthy that other biocompatible solid acids, such as citric acid and vitamin C, could effectively replace the BTCA, possibly offering a sustainable approach to silanized cellulose aerogels. The BC/BTCA/MTMS aerogel of cross-linked network exhibits mechanical elasticity, hydrophobicity, and rapid shape recovery. The mentioned properties are especially suitable for mechanical oil–water separation applications while also suggesting possibilities for thermal insulation uses [95]. In another report, the BC silylated bacterial cellulose aerogels (SBCAs) were prepared through the BC and MTMS through the CVD process, as represented in Figure 3a,b [96]. This process deposited a uniform hydrophobic and lipophilic polysiloxane layer onto the cellulose framework without forming obstructive particles within the pore structure. As a result, the porosity microarchitecture was maintained. The hybrid aerogels produced demonstrated exceptionally low densities ranging from 10 to 15 mg/cm^3^, remarkable porosity at approximately 99.1%, and decreased thermal conductivity between 27.3 and 29.2 mW/mK. Additionally, it demonstrated improved hydrophobic properties, with WCA exceeding 120°. Mechanically, the aerogels demonstrated excellent shear resistance, flexibility, and super-elasticity. As shown in Figure 3c,d, the SBCA effectively removes the dichloromethane and petroleum ether from underwater and on the water surface, respectively. As a multifunctional material, the aerogel showed highly effective oil absorbency with a maximum sorption capacity of ∼156 g/g (Figure 3e) and excellent reusability. Figure 3f,g represent the liquid density-dependent organic solvent sorption capabilities and absorption kinetics of SBCA. The aerogel has an extremely accessible porous structure that allows a high flux rate of 162 L/hg for continuous oil–water separation. Furthermore, the cyclic absorption capabilities of n-hexane in the SBCA are notably higher, as illustrated in Figure 3h [96].

**Figure 4:**
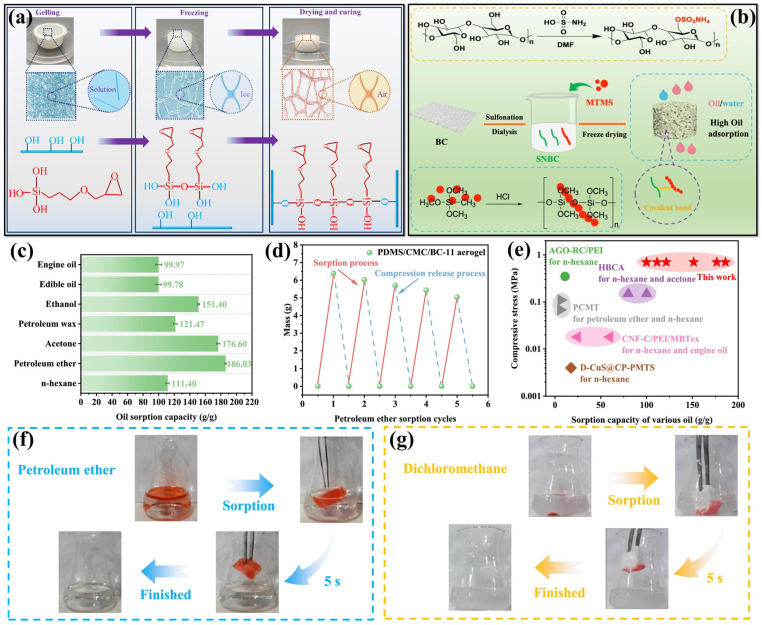
(**a**) Schematic of bacterial cellulose-based BK aerogel preparation and cross-linking method. Reprinted with permission from ref. [97]. Copyright © 2023 Elsevier B.V. (License number: 6086990264051). (**b**) Conceptual diagram of bacterial cellulose aerogel (HBCA) development process. Reprinted with permission from ref. [98]. Copyright © 2024 Elsevier B.V. (License number: 6086990407855). PDMS-CMC-BC aerogel: (**c**) absorption capacity of different oils, (**d**) petroleum ether sorption in 5 cycles, (**e**) compressive stress and sorption capacity compared with reported studies, (**f**) sorption capacity of petroleum ether on water, and (**g**) sorption capacity of dichloromethane underwater. Reprinted with permission from ref. [100]. Copyright © 2025 Elsevier B.V. (License number: 6086990560144).

The preparation of cellulose-based aerogels with greater mechanical stability via simple and eco-friendly methods remains a significant challenge. Hu et al. [97] developed a BC and γ-(2,3-epoxypropoxy)propyltrimethoxysilane trimethoxysilane(KH560) composite of BK aerogel through a green one-pot process involving in situ gelling and freeze-drying as schematically represented in Figure 4a. This method is significant as it does not require any chemical additives, which eliminates the necessity for post-synthesis washing. The aerogel generated demonstrated hydrophilic properties and proved effective for the sorption of cationic dyes. The aerogel exhibits a surface chemistry, which is easily adjustable for additional functionalization due to the presence of reactive hydroxyl and epoxy functional groups. For example, the hydrophobic modification resulted in a high-elasticity hydrophobic (HBK) aerogel exhibiting a WCA of 146.8°, which makes it appropriate for oil–water separation and allowing for recycling through mechanical squeezing. The aerogels demonstrated ultrahigh porosity (98.9–99.7%) and extremely low density (4.7–14.7 mg/cm^3^). In comparison to pristine BC aerogels, the BK aerogel displayed a 4-fold enhancement in elastic modulus, displaying an elastic recovery of 96.4% under 80% strain and noteworthy fatigue resistance. Moreover, the aerogel demonstrated the capacity to undergo immersion in water for several cycles of cationic dye sorption while maintaining its structural integrity, underscoring its durability and adaptability for environmental applications [97]. In another report, a hydrophobic BC-based aerogel (HBCA) was fabricated for efficient oil–water separation via a one-pot synthesis approach as shown in Figure 4b [98]. In this strategy, BC was first sulfonated and mechanically treated to obtain sulfonated nanofibrillated BC (SNBC) comprising of high electronegative properties. The in-situ polymerization was utilized for MTMS within the SNBC matrix and followed by freeze-drying, which resulted in a three-dimensional porous HBCA structure. The composite aerogel with 2 wt% MTMS exhibited remarkable hydrophobic properties, WCA of 152.4° and a specific surface area of 6.039 m^2^/g. The aerogel displayed outstanding selectivity and recyclability, efficiently sorbing a diverse array of oils and organic solvents. The ranging of 42.14g/g to 85.37g/g saturation capacity exhibited. Furthermore, the HBCA exhibited an impressive separation efficiency of 98.48% in continuous oil–water separation processes, highlighting the possibility of uses for industrial wastewater treatment and oil spill remediation [98]. A facile strategy was developed for an environmentally friendly BC-MXene aerogel for efficient crude oil-seawater separation [99]. Initially, silanized BC (SBC) aerogel was prepared via a silanization process using MTMS. Subsequently, MXene nanosheets were attached to the BC aerogel via a dip-coating technique for forming SBC-MXene aerogel. The aerogel underwent additional modification to improve its hydrophobic properties by immersing it in polydimethylsiloxane (PDMS). The aerogel attained as superhydrophobic and lipophilic aerogel with a CA of 136°. Owing to its exceptional photo-thermal and electro-thermal properties, the aerogel enabled rapid and selective absorption of high-viscosity crude oil from seawater under solar irradiation or applied voltage. Notably, the integration with a pump-assisted absorption device facilitated continuous and all-weather crude oil removal, employing the synergistic effects of solar irradiation and applied voltage, resulting in an impressive crude oil flux of 630 kg/m^2^h [99].The BC-based aerogel was developed by integrating PDMS and sodium carboxymethyl cellulose (CMC) via a facile freeze-drying approach for multifunctional environmental remediation [100]. As displayed in Figure 4d,e, the resulting CMC-BC and PDMS-CMC-BC aerogels exhibited notable mechanical strength (0.70 MPa at 80% strain), less density (0.097 g/cm^3^), and efficient high porosity (96%). The CMC-BC aerogel demonstrated excellent dye sorption capacity and outstanding oil sorption. The PDMS-CMC-BC-11 aerogel achieved rapid oil–water separation within 5 s (Figure 4f—petroleum ether, and Figure 4g—dichloromethane) and displayed outstanding oil sorption (186.03 g/g for petroleum ether) as represented in Figure 4c. The multifunctional aerogels offer a promising and sustainable approach for the removal of dyes and the separation of oil from water [100].

## 3. Cellulose with Different Kinds of Biopolymer Aerogels

Cellulose-based aerogels in combination with other biopolymers exhibited a promising material with sustainable and enhanced characteristics for applications in oil–water separation. Cellulose combined with additional biopolymers such as chitosan-, alginate-, lignin-, or gelatin-cellulose aerogels demonstrated increased efficiency and a broader range of applications in addition to its biodegradability, light weight, and porous structure. Hence, the chitosan enhances the antimicrobial and sorption properties in cellulose–chitosan aerogels for oil–water separation. Similarly, the incorporation of lignin into cellulose-lignin aerogels attributed the increase in hydrophobicity and mechanical strength, which in turn improves the structural stability and oil absorption efficacy of the aerogels. Moreover, gelation control and water retention are enhanced by alginate-cellulose aerogels, which are advantageous in controlled release systems and medical dressings. These hybrid aerogels are attractive alternatives to synthetic materials in eco-friendly applications due to their biodegradable, green components, which allow for tunable surface chemistry, porosity, and reusability.

### 3.1. Chitosan-Cellulose Based Aerogel

**Figure 5:**
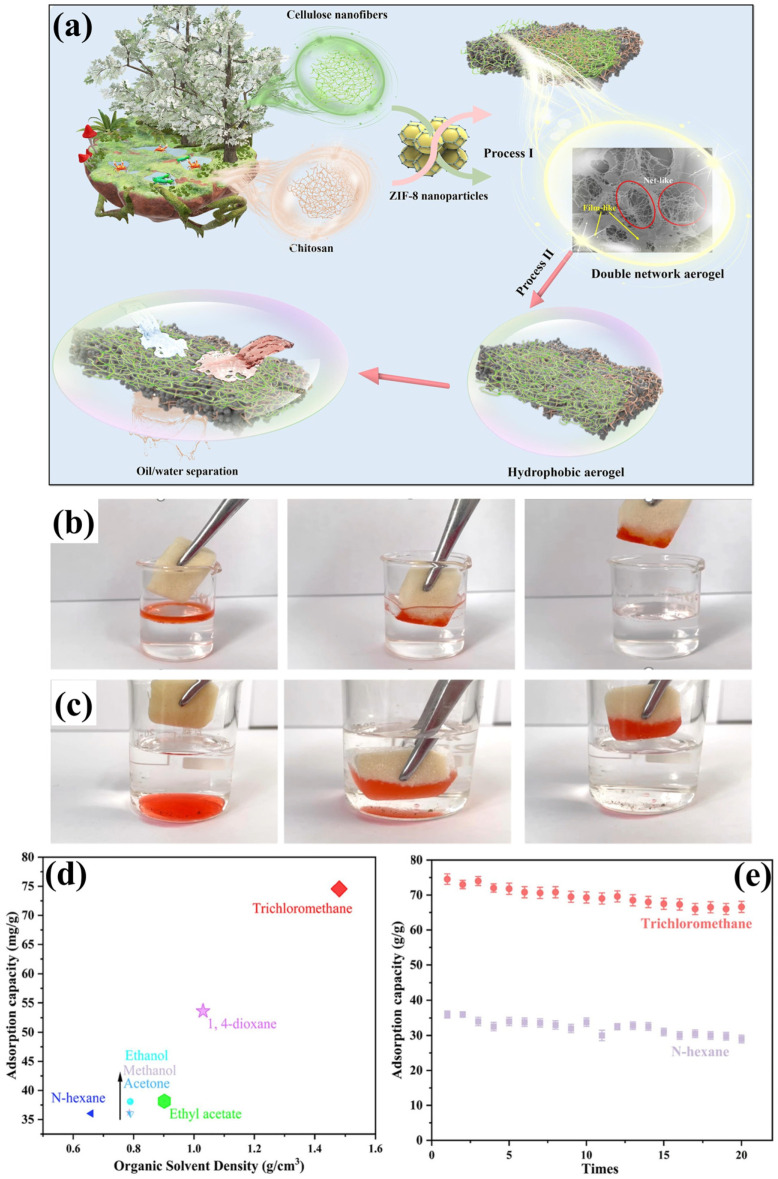
(**a**) Development steps for hydrophobic CNF/Chitosan-ZIF8 (CCZAn) aerogel. CCZAn aerogel (**b**) n-hexane sorption from the water surface, (**c**) trichloromethane sorption from underwater, (**d**) different densities of organic pollutants absorption capacity, and (**e**) cycle performance. Copyright ©. Reprinted with permission from ref. [105]. Copyright © 2023 Elsevier B.V. (License number: 6086991478239).

For oil–water separation, chitosan-based aerogels provide a number of benefits due to their high efficiency and ecological sustainability. The remarkable oil absorption properties of nearly 100 g of oil per gram of aerogel are attributed to their ultra-lightweight and highly porous structure. Additionally, the possible surface modifications have rendered them oleophilic and hydrophobic, facilitating selective oil separation from water. Chitosan (CS) is sourced from chitin, a naturally occurring biopolymer found in such things as crab shells, exoskeletons of shrimp, and lobster. CS serves as an eco-friendly alternative to conventional synthetic sorbents due to its biodegradable, renewable, and non-toxic properties [101]. Furthermore, to improve separation efficiency and facilitate recovery and reuse, CS aerogels can be modified with various functional properties of materials such as graphene oxide (GO) or magnetic nanoparticles (NPs). The advantageous characteristics of CS-containing aerogels are particularly attractive for applications in oil spill remediation and industrial wastewater treatment. A highly effective salt-tolerant superoleophilic aerogel was created by combining nanofibrillated cellulose (NFC) with CS in order to separate oil/water emulsions in marine environments [102]. The as-developed aerogel (CS/NFC) has the low density of 18.6 mg/cm^3^ with ultrahigh porosity of 98.7%. Moreover, the CS/NFC aerogel contains numerous irregularly shaped micropores with rough surfaces. These rough surfaces are responsible for enhanced underwater superoleophobicity (>150°) of CS/NFC aerogel. Furthermore, the electrostatic interactions and hydrogen bonding network between CS (amino –NH_2_) and NFC (–OH groups) increase the mechanical stability of aerogel for excellent reusability (>40 cycles) with significant separation efficiency (99%) [102]. Antimicrobial properties of microporous cellulose-based aerogel have been developed through facile cross-linking with CS using glutaraldehyde (GA) and the incorporation of quaternarized N-halamine siloxane (QHS) monomer. The as-prepared aerogel showed significant high porosity of over 97.66% with increased underwater OCA of 141.1° ± 2.1°, attributed to its higher hydrophilicity. Furthermore, the aerogel exhibited higher separation efficiencies of over 99.9% in oil/water separation. The use of QHS in aerogel enhances its exceptional antibacterial characteristics [103]. Zhang et al. [104] developed NFC/CS composite aerogel with distinct pore architecture through the soft-ice freezing method for enhancing the mechanical behavior (87.16 kPa under 80% strain) of aerogel. Moreover, the NFC/CS composite aerogel resulted in a higher specific surface area (58.095 m^2^/g), a smaller average pore size (5.75 nm), increased porosity (99.46%), and a lower density (0.008 g/cm^3^). The hydrating layer is created by strong inter- and intramolecular hydrogen bonding between the water and aerogel due to the presence of –OH groups in the aerogel. This resulted in the formation of rapid repulsive force between oils, which exhibited the core underwater superoleophobicity (>150°). Thus, the as-prepared NFC/CS aerogel showed 0.817 L/m^2^h (oil/water) and 0.826 L/m^2^h (oil/seawater) separations with 99.96% separation efficiency even after 20 cycles of separation [104]. A hydrophobic double-network CCZA aerogel (ZIF-8@CNF-CS) was developed by incorporating high-surface-area, highly crystalline ZIF-8 nanosheets into a CNF-CS matrix as schematically represented in Figure 5a [105]. Moreover, the MTMS was used to hydrophobically functionalize the aerogel surface. The as-prepared Si-CCZAs were found to have a significant porosity of 99.01% with an ultralight density of 15.87 mg/cm^3^. Moreover, it contains the specific surface area of 5.51 m^2^/g with average pore sizes of 4.32 nm. Si-CCZAs demonstrated an improved hydrophobicity of 132.6°, along with increased rigidity and recyclability achieved through hydrophobic modification. Hence, the Si-CCZAs attained 10,800 to 13,100 L m^−2^ h^−1^ flux with 99.8% separation efficiency due to their hydrophobic, double-network, and highly porous architecture. Moreover, a well-defined three-dimensional porous architecture facilitated a high sorption capacity for organic solvents, ranging from 35.99 to 74.55 g/g (Figure 5d). CCZAn aerogel showed the effective sorption of n-hexane from the water surface (Figure 5b) and trichloromethane from underwater (Figure 5c). Over 20 continuous cycles (Figure 5e), it exhibits excellent cyclic stability of 88% sorption capacity [105].

**Figure 6:**
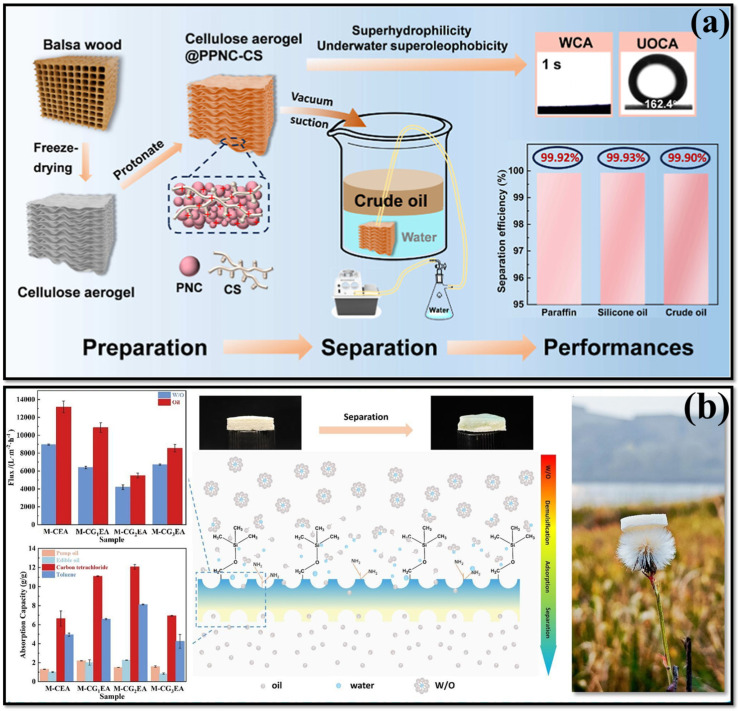
(**a**) Preparation and performance results of wood-based cellulose aerogel coated with a protonated nanocomposite chitosan coating. Reprinted with permission from ref. [106]. Copyright © 2024, American Chemical Society. (License number: 6087000159110). (**b**) The separation mechanism, flux, and sorption capacity of polyamidoamine modified chitosan/cellulose aerogel. Reprinted with permission from ref. [108]. Copyright © 2025 Published by Elsevier B.V. (License number: 6087000328593).

Recently, Huang et al. [106] developed a protonated nanocomposite chitosan-coated cellulose aerogel (cellulose aerogel@PPNC-CS) for high-viscosity oil/water separation as represented in Figure 6a. The process involved removing hemicellulose and lignin from balsa wood to produce a cellulose aerogel via freeze-drying, which was subsequently coated with a polymeric nanocross-linked chitosan (PNC-CS) network formed through a 1,4-conjugate addition reaction between dipentaerythritol pentaacrylate (DPPA) and branched polyethyleneimine (bPEI), cross-linked with CS. The final material was protonated by immersion in hydrochloric acid to yield cellulose aerogel@PPNC-CS. Thus, the cellulose aerogel@PPNC-CS exhibited increased underwater OCA (160°) with increased flux value of 1.391 × 10^5^ L/m^2^h, while maintaining a separation efficiency of >99.90% for high-viscous oil/water separation (Figure 6a) [106]. In order to improve mechanical robustness and structure, glycerol (GL) was added to a low-cost, bio-based CNF and CS combined aerogel [107]. Additionally, it was modified with using GA crosslinking and polymethyltrimethoxysilane (PMTS). The resulting CNF/CSA-PMTS aerogel displayed a covalently linked porous network with exceptional durability (78% stress retention after 80 cycles at 80% strain) and super elasticity (90% height retention post-compression). The aerogel displayed remarkable superhydrophobicity, featuring a WCA of 141°. The high porosity provide as efficient oil–water separation abilities, with absorption capacity reaching up to 65 g/g. It also provide 99.7% a separation efficiency. Moreover, the flux results were 18,000~28,000 L/m^2^ h for the CNF/CSA-PTMS. Based on reusability (after 10th cycles), the as-prepared aerogel retained absorption capacity of nearly 91.6% and achieved separation efficiency of 99.3% [107]. Recently, CS with cellulose-based polyamidoamine (PAMAM) aerogels has been developed by sequential cross-linking process [108]. In order to modifying the as-prepared aerogels into hydrophobic, CVD and silicification process using MTMS has been performed. The as-modified aerogel exhibited ultralight density (Figure 6b) of 0.06 to 0.11 g/cm^3^ with pore size ranging from ~123 to 125 nm. Moreover, the aerogel displayed the highest surface area of 1.93 to 12.56 m^2^/g with enhanced hydrophobicity of 139.5°. Furthermore, the as-developed aerogel accomplished increased flux values for water/oil emulsions (4198.60 Lm^−2^h^−1^) and carbon tetrachloride/water mixtures (5501.85 Lm^−2^h^−1^), as displayed in Figure 6b. The separation process was attributed to the dendritic amino group and the hydrophobic nature of the aerogel, as well as the varying surface energy levels in the aerogel resulting from the grafting of Si–CH_3_–groups (Figure 6b). This behavior effectively captured water droplets within the aerogel structure and facilitated the smooth removal of oil droplets [108]. The advanced, eco-friendly, and hydrophobic aerogel of CS containing cellulose has been developed through various methods, which demonstrate notable mechanical stability and separation efficiency for the treatment of oily wastewater.

### 3.2. Lignin-Cellulose Based Aerogels

**Figure 7:**
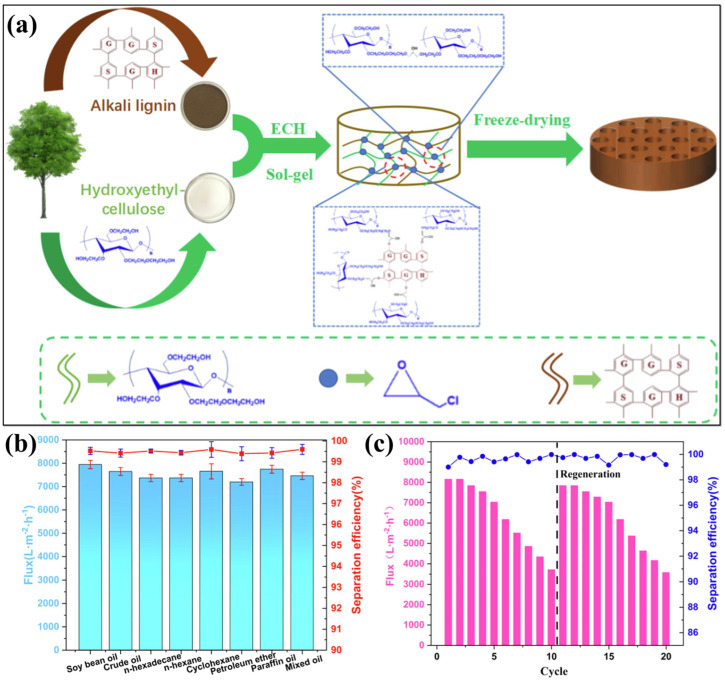
(**a**) Illustration of alkaline lignin into hydroxyethyl-cellulose (AL-HEC) preparation. AL-HEC-based aerogel: (**b**) various oil separation performance and (**c**) cyclic performance. Reprinted with permission from ref. [63]. Copyright © 2022 Elsevier Inc. (License number: 6087000494090).

An attractive option for oil–water separation is lignin containing aerogel, which is abundant, renewable, and biodegradable. Moreover, lignin is a major waste product of the paper and biofuel industries. Lignin possesses an extensive number of aromatic and functional groups, which can be modified to achieve hydrophobic and oleophilic characteristics for targeted oil absorption. The lightweight framework, porosity, and biological source of lignin provide an efficient and environmentally friendly alternative for oil–water separation. Aerogels developed using lignin exhibit enhanced oil absorption properties and can be chemically modified or combined with other materials such as cellulose to improve the mechanical strength and reusability. In addition, lignin aerogels made using a green synthesis method are a less harmful option than synthetic sorbents for cleaning up oil spills and other effluents. Tan et al. [63] developed three-dimensional interconnected lignin/cellulose aerogel via the sol–gel method using the freeze-drying technique with epichlorhydrin (ECH) as a cross-linking agent, as schematically represented in Figure 7a. The interconnected three-dimensionality of the as-prepared aerogel was attributed to the cross-linking agent (ECH) that enhances its structural support. Furthermore, the lignin introduction into cellulose aerogel enhances its underwater superoleophilicity, surface roughness, and water permeation speed, along with controlling the pore size for regulating the separation flux. As shown in Figure 7b, the as-developed aerogel demonstrated excellent separation efficiency for a range of oil–water mixtures and oil-in-water emulsions, exhibiting a high separation flux of 7646 ± 167 L/m^2^ h and an oil rejection rate exceeding 99%. Moreover, the AL-HEC represents the considerable cycle performance (Figure 7c) [63]. To enhance the performance of cellulose-based aerogels for oil/water separation, the sol–gel process followed by freeze-drying was utilized to develop the hydrophobic and magnetic hydroxyethyl cellulose–lignin (CL) aerogel [109]. To enhance the hydrophobicity and magnetic responsiveness of CL (HMCL) aerogel, an ultrasonically treated process with Fe_3_O_4_ NPs and n-dodecyl mercaptan was applied. A 3D porous architecture was established by the addition of lignin, which significantly improved the mechanical stability of the aerogel. The presence of Fe_3_O_4_ in aerogel improved the reusability during a continuous cycle of oil–water separation by enabling magnetic property. The modified aerogel exhibited superior mechanical strength of 54.26 kPa (at 80% strain) and an ultralow density ranging from 0.0443 to 0.0718 g/cm. Moreover, it showed a high sorption capacity between 18.92 and 32.11 g/g and an improved oil/water separation flux of 2986 L/m^2^·h [109]. In certain cases, nanocellulose showed low lipophilicity and constrained mechanical strength, which is a challenge for practical applicability. The lignin-containing cellulose aerogel (LCMA) featured a robust three-dimensional porous network, improving both structural integrity and oil absorption capacity [110]. Directional freezing further induced an aligned pore structure (DF-LCMA), which imparted anisotropic mechanical properties. Subsequent hydrophobic modification via thermal CVD of methyltrichlorosilane (MTCS) yielded a superhydrophobic variant (S-DF-LCMA). The aerogel demonstrated an increased porosity of 98.87%, a density of 8.3 mg/cm^3^, and WCA of 168°, which are critical for lightweight and high-capacity absorption applications. The lignin incorporation increased the oil absorption capacity (by 8 to 12 times) and compressive modulus (by 2036%) compared to pure cellulose aerogels, which showed its potential for oil–water separation [110].

### 3.3. Alginate-Cellulose Based Aerogels

**Figure 8:**
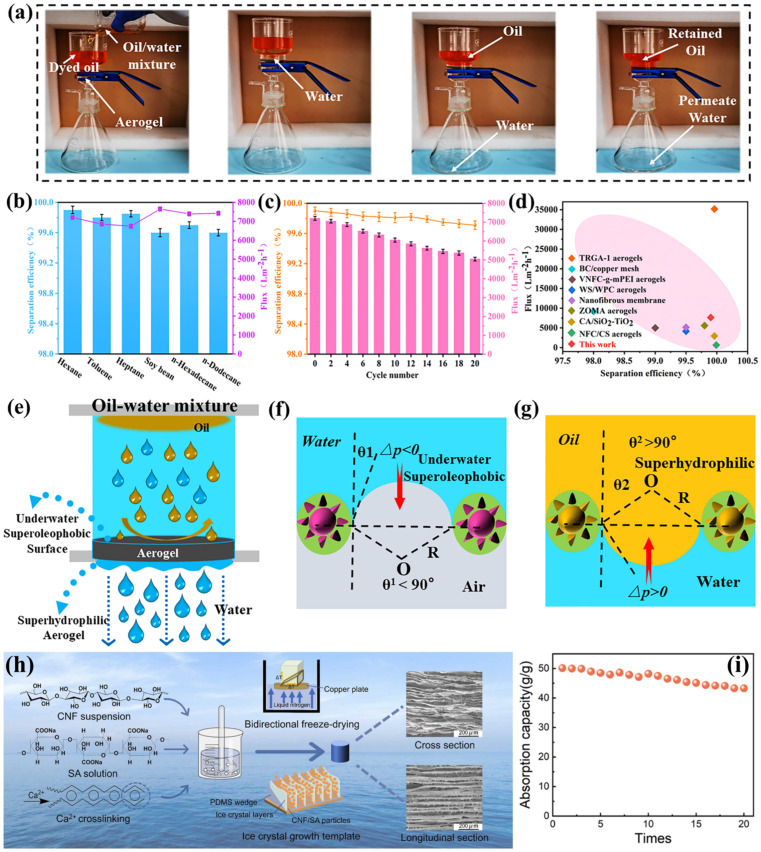
CMC/Sodium Alginate@TiO_2_−MWCNTs (CSTM) aerogel: (**a**) gravity-driven oil–water separation. Separation efficiency and flux of CSTM aerogel (**b**) different oils, (**c**) 20-cycle performance, and (**d**) comparative with reported studies. (**e**) Possible oil–water separation mechanisms via CSTM aerogel. (**f**,**g**) Modeling of superhydrophilic oil-viscosity resistance. Reprinted with permission from ref. [113]. Copyright © 2024 Elsevier Ltd. (License number: 6087000689880). Cellulose nanofibers/alginate (CNF-SA) aerogels: (**h**) preparation processes and (**i**) recoverability. Reprinted with permission from ref. [114]. Copyright © 2022 Elsevier B.V. (License number: 6087000922308).

Sodium alginate (SA) is a marine-derived anionic polysaccharide made up of mannuronic and guluronic acid. The combination of SA and cellulose can produce low-density aerogels with porosities ranging from 94% to 97%. These aerogels demonstrate integrated macropores and mesopores, which contribute to their oil-uptaking capabilities by establishing capillary forces and absorption behavior [111,112]. Moreover, the composite aerogels are biodegradable, renewable, and biocompatible with enhanced porosity, mechanical stability, and reusability that represent the suitable alternative for synthetic sorbents. Wang et al. [113] fabricated superhydrophilic carboxymethyl cellulose-based composite aerogels (CMC-SA@TiO_2_-MWCNTs, CSTM) via ionic cross-linking and facile freeze-drying approaches for enhancing the oil/water separation. The incorporation of TiO_2_/MWCNTs nanocomposites into polysaccharide aerogels enhances the underwater oleophilic and mechanical stability of composite aerogels. The as-prepared aerogel showed ultra-low density (0.0326 g/cm^3^), high porosity (98.8%), increased specific surface area (168.1 m^2^/g), along with enhanced mechanical stability of 2.688 MPa. The CSTM aerogel showed the efficient gravity drive oil–water separation, as shown in Figure 8a. A separation efficiency of 99.9% has been observed for immiscible oil–water combinations, with a high separation flux of around 7650 L/m^2^ h. Up to 99.3% separation efficiency and a flow of 3952 L/m^2^h were obtained for the surfactant-stabilized oil-in-water emulsion. As represented in Figure 8b–d, the CSTM aerogel showed efficient separation efficiency, flux, and cycle performance. Figure 8e–g displayed the possible oil–water separation mechanisms via CSTM aerogel and modeling of superhydrophilic oil-viscosity resistance [113]. A 3D-lamellar hydrophobic aerogel based on CNF and SA was fabricated through bidirectional freeze-drying, chemical cross-linking using D-(+)-gluconic acid δ-lactone and CaCO_3_, followed by surface modification with MTMS as schematically illustrated in Figure 8h [114]. The resulting CNF-SA aerogel exhibited enhanced hydrophobicity (~144.5° WCA), greater porosity of 97.85%, and low density of 24.2 mg/cm^3^. In addition, it has the ability to efficiently separate oils and water, as shown by its significant oil sorption capabilities (41.16 to 88.91 g/g) and efficient recoverability (Figure 8i) [114].

## 4. Cellulose with Various Synthetic Polymer Aerogels

### 4.1. PVA-Cellulose Based Aerogels

**Figure 9:**
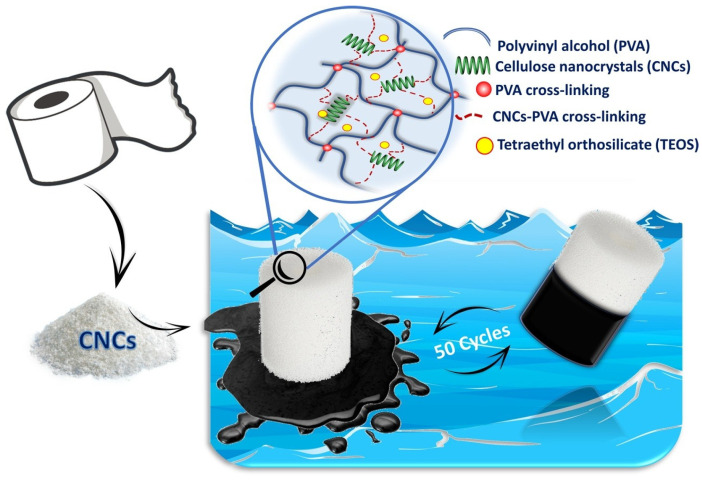
Schematic representation of the internal connectivity and role of CNCs/PVA/TEOS aerogel during the oil–water separation. Reprinted with permission from ref. [118]. Copyright © 2021 Institution of Chemical Engineers. Published by Elsevier B.V. (License number: 6087001073179).

The combination of mechanical stability, adjustable porosity, and ecological compatibility has established polyvinyl alcohol (PVA)-based cellulose aerogels as an important component for oil–water separation [115]. The synthetic and biodegradable properties of PVA mixed with cellulose further improve the stability, structural integrity, and flexibility of aerogels [116,117]. The possible generation of hydrogen bonding between cellulose and PVA chains creates a robust aerogel matrix. Surface treatments such as silanization or nanoparticle inclusion may change and provide superhydrophobic and superoleophilic properties. These modified PVA-cellulose aerogels exhibit remarkable selectivity in removing oil from water and oil absorption capabilities. In addition, it is highly reusable throughout a wide range of absorption-desorption cycles, which showed it as viable for oil spill cleanup and industrial wastewater treatment. Their eco-friendliness and biodegradability provide a promising long-term substitute for traditional oil sorbents. A superhydrophobic and eco-friendly aerogel was developed from cellulose nanocrystals (CNC) obtained from waste tissue paper and PVA (Figure 9) [118]. Moreover, the hydrolyzed tetraethyl orthosilicate (TEOS) was utilized for the aerogel modifications via a silylation-assisted freeze-drying process. The CNC-PVA-TEOS aerogel exhibited a superhydrophobic surface with a water contact angle of 154.93° ± 4.14°, a low density of 0.017 g/cm^3^, and a remarkable porosity of 98.42% with an outstanding surface area of 76 m^2^/g. Despite subjecting the aerogel to 20 cycles of sorption and mechanical squeezing, it maintained 92% of its original sorption performance. Moreover, it demonstrates an outstanding sorption capacity ranging from 69 to 168 g/g for different oils and organic solvents. The CNC-PVA-TEOS aerogel exhibited higher flux, about 8321 L/m^2^h. Furthermore, it demonstrated exceptional mechanical behavior by recovering 89% of its original form after 50 compression cycles [118]. In another study, Yu et al. [119] used chemical cross-linking, freeze-drying and silanization techniques for fabricating cellulose-PVA composite aerogel. The composite aerogel demonstrated remarkable mechanical stability, which was attributed to the combined impact of hydrogen bonds and chemical cross-linking. It has been observed that aerogel showed 490.7 kPa of compressive stress displayed at 90% strain. Moreover, the cellulose-PVA aerogel displayed excellent superhydrophobic behavior (156.6° WCA). Furthermore, in water/cyclohexane emulsions the aerogel exhibited enhanced permeation flux of 7176.3 L/m^2^h with 4550.6 L/m^2^ of total separation [119].

**Figure 10:**
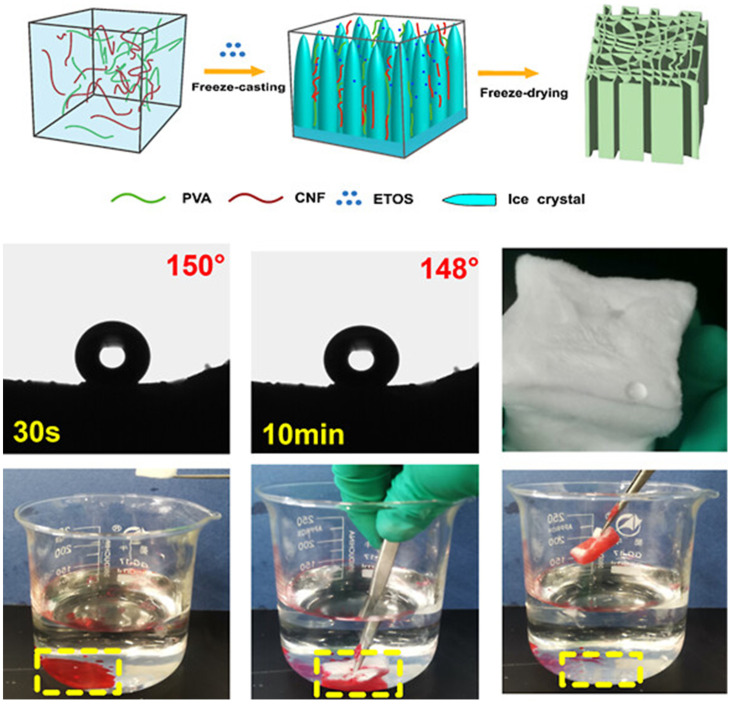
PVA-CNF-ETOS Elastic Aerogel: fabrication steps, WCA at 3 s and 10 min, and selectively removes the chloroform. Reprinted with permission from ref. [120]. Copyright © 2023, American Chemical Society. (License number: 6087001233570).

Chen et al. [120] developed anisotropic nanocellulose-based aerogels featuring a lamellar structure of PVA and CNF combination as schematically represented in Figure 10. Hydrophobic modification of PVA-CNF is achieved through the hydrolysis of ethyl trimethoxysilane (ETOS). The resulting ETOS-modified PVA-CNF aerogels exhibited an ultralow density of 10.8 kg/m^3^, where PVA-CNF alone is 6.5 kg/m^3^. Moreover, it shows excellent compressive resilience and hydrophobic WCA of 148°, and enhanced porosity of 98.4%. The selective oil absorption of PVA-CNF-ETOS ranges from 50 to 110 g/g. This aerogel has many potential applications, including environmental cleanup, surgical instrument distribution, and pharmaceuticals, because of its strong affinity for alcohols and lipids [120]. In another study, a hierarchically structured CNF-PVA-PDSO hybrid aerogel was developed through polymer mineralization and interfacial engineering strategies [121]. Coating the nanocellulose network with mineralizers containing long-chain hydrophobic alkyl groups improved the porosity architecture, which was achieved by building biopolymeric units and strengthening the cellulose framework by silanization. Directional freeze-drying and acid hydrolysis of DTOS enabled multiscale interaction and wetting-induced mineralization, forming layered hybrid interfaces. The resulting aerogel exhibited 11.4 kg/m^3^ density, high porosity (99.2%), excellent mechanical properties, and rapid recovery under high strain. It maintained structural integrity after many compression cycles and displayed superhydrophobicity with a WCA of 152°. The aerogel demonstrated high thermal stability with only 44.47% mass loss at 700 °C. The aerogel showed a high absorption capacity (67 to 133 g/g) and retained 57.9% of its capacity after 40 sorption cycles. The improved mechanical, thermal, and sorption performance of the CNF-PVA-PDSO aerogels compared to traditional cellulose-based aerogels highlights its promise for thermal insulation and environmental remediation [121]. A superhydrophobic/superoleophilic MTCS-modified PVA/cellulose (MPCA) aerogel was fabricated with an asymmetric structure [122]. The MPCA demonstrated a compact microporous upper layer and a honeycomb-structured macroporous lower layer, facilitating targeted oil absorption and emulsion separation. The aerogel exhibited high oil absorption capacity (37.2 g/g to 92.4 g/g), excellent separation efficiency (99.5%), and high separation flux (631.9 to 2368.7 L/m^2^h). The aerogel, enhanced with GO, exhibited a light absorption rate of 94.8%, enabling quick solar-to-thermal conversion and reaching surface temperatures of up to 62.5 °C, which is effective for high-viscosity crude oil sorption. In solar-driven desalination applications, the oxygen plasma-treated MPCA demonstrated remarkable superhydrophilicity, achieving an impressive evaporation rate of 1.39 kg/m^2^h and a solar energy conversion efficiency of 89.7%. Additionally, it maintained self-cleaning salt resistance, with complete dissolution of accumulated salt occurring within 20 min. The multifunctional properties of MPCA exhibit an efficient oil–water separation and sustainable freshwater production [122]. Qu et al. [123] reported the fabrication of a unique cellulose-based aerogel through the incorporation of UiO-66-NH_2_ (metal–organic frameworks, MOFs) into a PVA-CNF matrix (CPU/A). The CPU/A demonstrated outstanding physicochemical properties in comparison to the pristine PVA/CNF aerogel. The incorporation of UiO-66-NH_2_ increased the pore volume and mechanical stability and reduced the pore size along with increasing the specific surface area. Among the different composites, the UiO-66-NH_2_ of 10 wt% in the CPU/A-0.10 aerogel exhibited the higher performance with enhanced formaldehyde sorption capacity and oil–water separation efficiency. Furthermore, the aerogel demonstrated outstanding reusability, preserving both its structural and functional integrity during several cycles of sorption and desorption [123]. Recently, the combination of CMC, PVA, and chemically modified dodecyltrimethoxysilane (DTMS)-derived silica (SiO_2_) NPs has been fabricated as hydrophobic and lipophilic cellulose-based CP/SiO_2_@Fe^2+^ composite aerogels [124]. The addition of Fe^2+^ ions and GA acted as dual crosslinking agents, playing a crucial role in maintaining the structural integrity and improving the functional performance of the aerogel. The increased porosity (98.60%), lesser density (0.021 g/cm^3^), enhanced specific surface area (132.13 m^2^/g), and higher WCA (139°) were attained by the CP/SiO_2_@Fe^2+^ composite aerogel. The inherent characteristics contribute to achieving a higher oil absorption capacity (99.9%). The controlled release of Fe^2+^ ions at the oil–water interface facilitates the localized activation of persulfates, which improves the targeted production of SO_4_•^−^ radicals. This interfacial activation strategy successfully mitigated radical quenching, leading to improved stability and reactivity of the produced oxidative species. The elevated presence of radicals at the interface enabled effective oxidative demulsification of oil and water emulsions. Additionally, the aerogel demonstrated a high permeation flux of 19,130 L/m^2^h with a separation efficiency exceeding 99% [124].

### 4.2. PEI-Cellulose Based Aerogels

**Figure 11:**
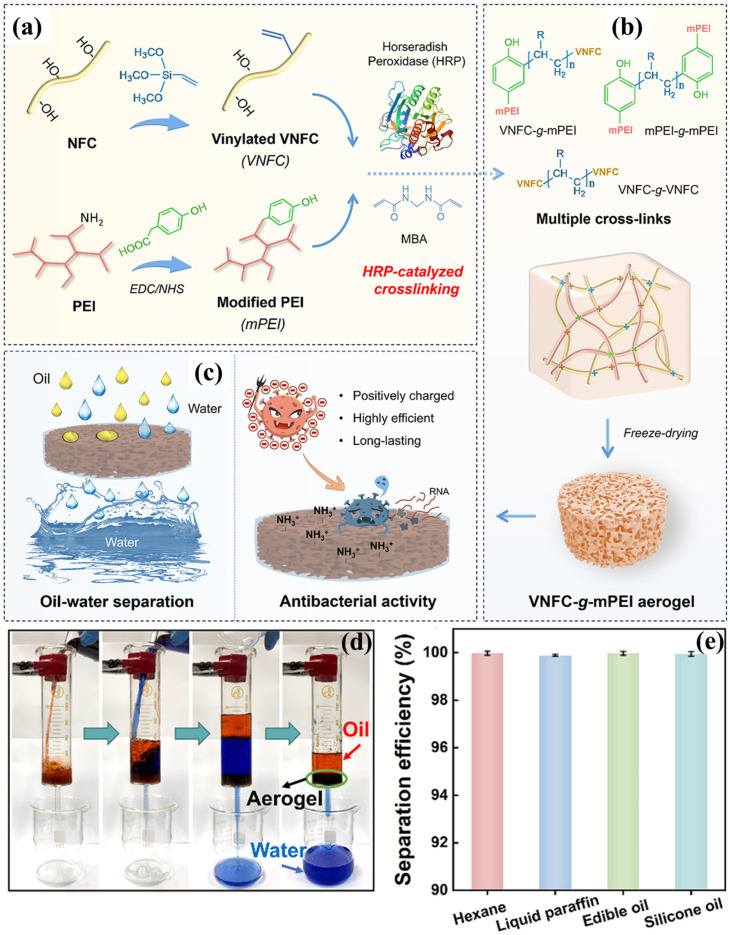
VNFC-g-mPEI Aerogel: (**a**) modification of NFC and PEI, (**b**) preparation steps of VNFC-g-mPEI Aerogel, (**c**) possible mechanism of antimicrobial activity and oil/water separation, (**d**) oil–water mixture separation through filtration, and (**e**) separation efficiency. Reprinted with permission from ref. [126]. Copyright © 2023, American Chemical Society. (License number: 6087001420167).

Polyethyleneimine (PEI) contains a high concentration of amine groups, which contributes to its significant surface reactivity and adjustable wettability. Furthermore, PEI improves the structural integrity of cellulose aerogels and creates numerous active sites for additional chemical modifications, including hydrophobization and nanoparticle incorporation. This leads to the development of aerogels that are both superhydrophobic and superoleophilic, capable of absorbing oil while effectively repelling water. Moreover, the incorporation of PEI into the aerogel significantly improves its stability under harsh environmental conditions, which improves its reusability across multiple separation cycles. The eco-friendliness of cellulose combined with the functional adaptability of PEI makes these types of aerogels suitable for oil spill cleanup and industrial wastewater treatment. Superhydrophilic composite NFC-based aerogel developed through graft polymerization and cross-linking showed the benefit for oil/water separation [125]. The NFC was oxidized using the TEMPO oxidation method, and oxidized NFC has been graft polymerized in the presence of 3-(3′-acrylicacidpropylester)-5,5-dimethyl hydantoin (APDMH) to obtain poly(APDMH)-g-ONFC (PAC). The 3-glycidoxypropyltrimethoxysilane (GPTMS) utilized for chemical cross-linking between PAC and PEI as PAC-g-PEI aerogel. In order to achieve the antimicrobial properties of PAC-g-PEI aerogel, it has been chlorinated with NaClO to obtain PAC-g-PEI-Cl aerogel. The PAC-g-PEI aerogel exhibited a density of 67 mg/cm^3^ with an enhanced porosity of 94%. The superhydrophilicity (WCA = 0°) nature of aerogel showed enhanced squeezability and flexibility in water, increasing the performance of oil/water separation. After 50 cycles of oil/water separation, the aerogel showed the greater flux (>9500 L/m^2^ h) with 99% separation efficiency. Moreover, the PAC-g-PEI-Cl aerogel showed an antimicrobial effect for inhibiting the growth of biofilm during oil/water separation [125]. Eco-friendly options have been investigated for the production of NFC-based aerogels to address the challenges associated with traditional NFC aerogels. Fan et al. [126] prepared antibacterial NFC-based aerogels via an enzymatic cross-linking strategy. The vinylated NFC (VNFC) and modified PEI (mPEI) developed through introducing vinyl and phenolic hydroxyl groups onto NFC and PEI, respectively, as expressed in Figure 11a. Cross-linking between VNFC and mPEI was catalyzed by horseradish peroxidase through free-radical coupling (Figure 11b). The N,N′-methylenebisacrylamide is utilized to reduce steric hindrance and enhance network formation. The VNFC–g–mPEI aerogel showed 55.1 mg/cm^3^ of density and 95.5% of porosity and outstanding mechanical stability in both wet and dry environments. An underwater oil contact angle of over 140° was achieved because of the exhibition of exceptional superhydrophilic and underwater oleophobic properties. It demonstrated a great potential for sustainable oil–water separation (Figure 11d) by maintaining a 99% separation efficiency (Figure 11e) and 5000 L/m^2^ h flux after 50 cycles. The VNFC-g-mPEI can provide efficient oil–water separation together with high anti-microbial activity as schematically represented in Figure 11c [126]. The same research group established the oxidized NFC-based aerogel with a multi-network cross-linked structure designated as C-g-PEI-PMTS [127]. Ethylene glycol diglycidyl ether served as a bifunctional cross-linker and promoted self-crosslinking among oxidized NFC and PEI chains. Moreover, PMTS was introduced for hydrophobic modification of aerogel, yielding a surface-grafted aerogel with enhanced interfacial strength. The C-g-PEI-PMTS aerogel exhibited high porosity of 95.73% and ultralow density of 53.80 mg/cm^3^. Moreover, it exhibits substantial elasticity of 95.86% recovery with a hydrophobic WCA of 130.0°. It retained more than 96% capacity after 20 compression-sorption cycles, demonstrating great recyclability and robust oil sorption performance. Furthermore, the aerogel maintained >99% separation efficiency and a high permeation flux (~5000 L/m^2^ h) for trichloromethane–water mixtures over 50 reuse cycles [127].

A multifunctional superhydrophilic–oleophobic (SHI–OP) CPF composite aerogel has been developed through a one-pot condensation approach via incorporating fluorinated surfactant (FS-60), PEI, CNFs, and GPTMS [128]. The 3D architecture has received recognition from CNF, while the presence of significant amine groups in PEI contributes to its hydrophilic characteristics and electrostatic interactions. The FS-60 component, which consists of hydrophilic moieties and oleophobic fluorinated chains, provides the aerogels wettability. The aerogels are ultralight density (0.0256 g/cm^3^) with high porosity (98.30%) and exhibit excellent mechanical resilience under cyclic compression. The as-developed CPF aerogels exhibited an increased permeation flux (>9060 L/m^2^h). Moreover, the presence of lamellar architecture with linked microporous networks exhibiting high oil–water separation efficiency (>99%). The multifunctional aerogels based on cellulose provide new options for improved water treatment applications, such as the separation of oil and water and the remediation of dyes [128]. In another report, Wu et al. [129] developed nanocellulose (NC)-based hydrophobic aerogels with MTMS modification. PEI and tannic acid (TA) were utilized as cross-linking agents, and aerogel was formed with CNF and TEMPO-oxidized CNF (TCNF). The TCNF-PEI aerogel showed a uniform laminated 3D porous framework. The maximum sorption behavior is 48.05, 44.67, 62.93, and 112.30 g/g for TCNF-TA, CNF-TA, CNF-PEI, and TCNF-PEI, respectively, where TCNF-PEI showed the considerably higher performance. Moreover, the enhanced porosity, surface area, and intricate network structure provide impressive recyclability (>74% after 5 cycles). The characteristics of TCNF-PEI aerogels establish them as durable and eco-friendly materials suitable for effective oil–water separation [129].

### 4.3. PDA-Cellulose Based Aerogel

**Figure 12:**
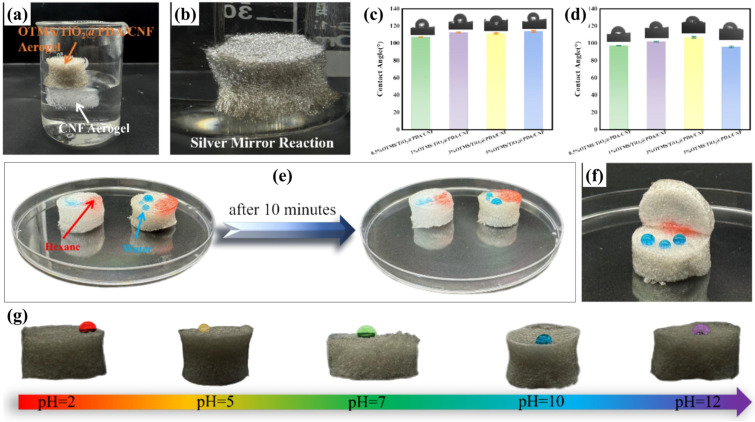
(**a**) Digital photograph of OTMS-TiO_2_@PDA-CNF and pristine CNF aerogels in water. OTMS-TiO_2_@PDA-CNF aerogels: (**b**) underwater immersion, (**c**) uncompressed, and (**d**) compressed optical contact angle. (**e**) OTMS-TiO_2_@PDA-CNF and pristine CNF aerogels wetting behavior results using water and hexane droplets. (**f**) Cross-sections and (**g**) surface wetting performance at various pH of OTMS-TiO_2_@PDA-CNF aerogel. Reprinted with permission from ref. [25]. Copyright © 2025 Elsevier B.V. (License number: 6087010110107).

Cellulose-based aerogels added with polydopamine (PDA) have garnered significant attention for oil–water separation due to their remarkable surface adhesion characteristics, adjustable wettability, and eco-friendly nature. A 3D Janus structure cellulose aerogel with multiple interlinked networks has been developed through an in situ approach [130]. Additionally, the hydrophobic modification on cellulose aerogel was performed by MTMS/trimethylchlorosilane. Furthermore, the in situ polymerization technology was utilized for PDA coating to develop a hydrophilicity on one side of aerogel. The uneven wettability observed on cellulose aerogel, where a hydrophobic surface (WCA = 142°) and an oleophilic side on one end, contrasted with a hydrophilic and underwater oleophobic surface on the opposite side. This unique configuration led to impressive results in high separation efficiency (99.5%) and permeation flux of 3121 L/m^2^ h [130]. Bi et al. [25] reported the development of a lightweight CNF-based aerogel incorporating TiO_2_@PDA core–shell NPs. The TiO_2_ has been enclosed by PDA through self-polymerization. The PDA and TiO_2_ have high adhesive capacity and the ability to attach strongly to the CNF matrix, respectively, which leads to the formation of a TiO_2_@PDA-CNF aerogel. Moreover, hydrophobic modification was carried out by introducing octadecyltrichlorosilane (OTMS) via vacuum infiltration, yielding a highly oleophilic and hydrophobic aerogel of OTMS-TiO_2_@PDA-CNF as displayed in Figure 12a. The underwater immersion, uncompressed and compressed optical contact angles of OTMS-TiO_2_@PDA-CNF aerogel are shown in Figure 12b, Figure 12c and Figure 12d, respectively. The as-developed aerogel exhibit with a WCA of 113.9°. The integration of TiO_2_@PDA significantly enhanced the mechanical robustness of the aerogel while increasing antimicrobial activity. The resulting OTMS-TiO_2_@PDA-CNF aerogel exhibited a higher porosity (87.7%) and lower density of 0.1171 g/mL. These properties can provide a large surface area for oil sorption (Figure 12e,f). Moreover, it showed outstanding absorption capabilities for a range of organic solvents and light and heavy oils, with a maximum uptake of 59.9 g/g. The surface wetting performance at various pH of OTMS-TiO_2_@PDA-CNF aerogel is shown in Figure 12g. Furthermore, the presence of TiO_2_ in aerogel enhanced the environmental adaptability by photocatalytic antimicrobial activity (99.14% sterilization rate) [25].

### 4.4. Cellulose with Other Polymers as Aerogels

A wide variety of different polymers, including polyurethane (PU) and polyethylene glycol (PEG), have been taken into consideration for the production of aerogels with cellulose for the purpose of oil–water separation. The PU served as an efficient material for various environmental remediation applications, specifically separation processes [131]. The pretreated cellulose and decafluorobiphenyl are utilized for modifying the PU foam (PUF), which enables enhanced oil–water separation [132]. The incorporation of Fe_3_O_4_ NPs into altered PUF exhibited increased magnetic sensitivity for aerogel recovery after numerous cycles along with high oil absorption capacity. The modified PUF aerogel exhibited high absorption capacity (ranging from 9 to 32 times its own weight). Moreover, it exhibits a high flux of up to 48,750 L/m^2^h for n-hexane. In the case of oil–water separation, the advantages include an exceptional separation efficiency of more than 97.68% and an enhanced reusability of up to fifty times [132]. In another report, Tang et al. developed a novel cellulose-based aerogel with dual wettable properties for water-in-oil and oil-in-water separation [38]. The CNF has been cross-linked with dicarboxylated PEG for obtaining CNF aerogel (CPM 600H). In order to obtain an aerogel with effective porosity and framework, the ratio of cross-linker to CNF has been tuned and optimized to be 1:4. The CPM 600H aerogel exhibited a 6.6 μm average pore diameter, 96.7% porosity, and a density of 31.5 mg/cm^3^. Moreover, the aerogel exhibited strong mechanical stability and showed an efficient recovery ratio after a few cycles. The CPM 600H aerogel showed the permeation flux of 5.80 L/m^2^ h, 496.48 L/m^2^h, and 1371.90 L/m^2^h for water-in-soybean oil, water-in-diesel, and water-in-hexane emulsion, respectively. It was shown that the increased superoleophilic qualities of aerogel were responsible for this increased permeation flux. Additionally, the size sieving and hydrophilic features of CPM 600H aerogel contributed to the increased oil/water separation [38].

## 5. Cellulose and Organic/Inorganic Material Composite Aerogels

**Figure 13:**
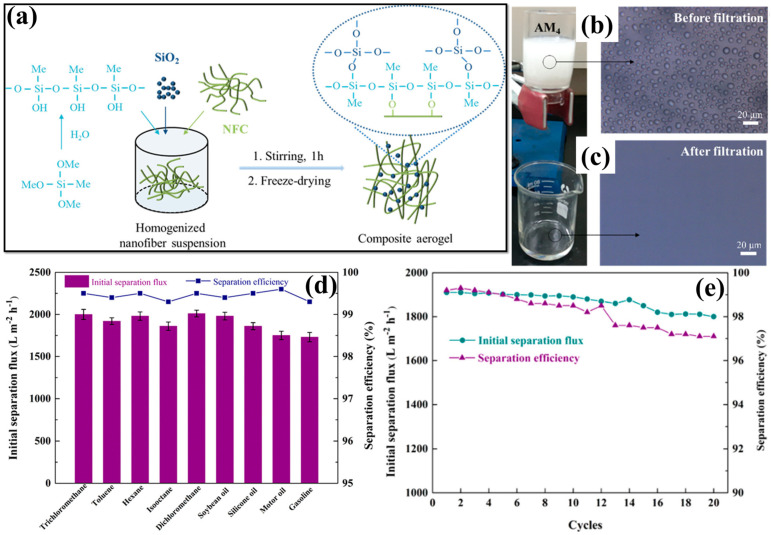
(**a**) Schematic of CNF-based CNF−MTMS with different loadings of SiO_2_ composite aerogel preparation. Gravity-driven separation of water-in-petroleum using CNF−MTMS−SiO_2_ aerogel: optical images of emulsions (**b**) before and (**c**) afterward filtration/separation. (**d**) Different water-in-oil emulsions and (**e**) water-in-petroleum ether emulsion separation up to 20 cycles using CNF−MTMS−SiO_2_ aerogel. Reprinted with permission from ref. [133]. Copyright © 2018, American Chemical Society. (License number: 6087010334353).

In addition to the combination of polymers, the hybrid combination of cellulose with inorganic materials demonstrated a significant amount of activity in the process of oil–water separation. A superhydrophobic CNF composite aerogel has been synthesized through homogenous combination of CNF with the desired amount of SiO_2_ and MTMS, as schematically illustrated in Figure 13a [133]. Both the porosity and the surface roughness of the aerogel are improved as a result of the incorporation of SiO_2_. Subsequently, MTMS actively participates in the hydrophobic alteration of the aerogel by lowering the surface energy. This leads to the modification of the aerogel. With a density of less than 6.43 mg/cm^3^, the composite aerogel showed an increased porosity of more than 99.6%. In addition to this, the aerogel demonstrated an increased superhydrophobicity of 168.4° (WCA). Its superoleophilic nature facilitates the penetration and transportation of oil into the aerogel, which is responsible for the effective separation of oil and water (Figure 13b,c). As an outcome of this, the composite aerogel demonstrated (Figure 13d) an enhanced flux of 1910 ± 60 L/m^2^h, achieving a separation efficiency of over 99% during the separation of water-oil emulsions stabilized by surfactant, even without the application of any external pressure. The high porosity and accelerated demulsification rate from the combination of superhydrophobic behavior with hierarchical porous assembly of composite aerogel were responsible for the accomplished goal of achieving the enhanced permeance flux. In addition, the superhydrophobic CNF composite aerogel demonstrated enhanced antifouling behavior and steady flux performance during the continuous cycle of oil–water separation (Figure 13e) in the energy management strategy [133]. Qiao et al. [134] developed a nanocellulose-based aerogel in both 3D bulk forms and 2D film-like forms to attain effective oil–water separation. The aerogel consists of elastic, superhydrophobic, and anisotrophic properties. The CNFs were obtained from wood pulp via TEMPO-mediated oxidation and subsequent high-pressure homogenization techniques. Through the process of directed freeze-drying of the CNF solution, an anisotrophic aerogel structure has been developed. PDMS was added into the aerogel by soaking and heat treatment processes. This was carried out in order to introduce superhydrophobic qualities into the formulation. The resulting CNF–PDMS aerogel exhibited a high WCA of 163.5°, an ultra-low density of 22.7 mg/cm^3^, and a robust anisotropic porous scaffold. Over a period of numerous cycles, this aerogel maintained an exceptional level of reusability and displayed significant oil absorption capabilities ranging from 24 to 48 g/g. Furthermore, continuous oil–water separation was accomplished by utilizing CNF–PDMS aerogel sheets in conjunction with gravity-driven filtration. This resulted in a high permeation flux of 2800 L/m^2^h and a separation efficiency of 99.9%. Moreover, block-shaped aerogels facilitated an immediate and efficient oil–water separation flux of 145 L/hg when subjected to vacuum suction. The results highlight the adaptability and scalability of anisotropic CNF–PDMS aerogels, positioning them as sophisticated sorbents for effective oil–water separation applications [134]. An efficient and eco-friendly approach to create hydrophobic cellulose-based aerogels made from CNFs, silylated castor oil (ICO), and tannic acid (TA), referred to as CNF-TA-ICO aerogels [135]. CO was subjected to a chemical modification process via utilization of 3-isocyanatopropyltriethoxysilane (IPTES) in order to generate ICO, which served as a hydrophobic component. After simultaneously depositing ICO and TA on the CNF matrix, a freeze-drying method was carried out in order to create a lightweight and porous aerogel with a surface chemistry. An extremely low density of 24.0 mg/cm^3^ along with a remarkable porosity of 98.32% was attained and also revealed a high WCA of 135.6°. Consequently, the resulting CNF-TA-ICO aerogel demonstrated an impressive oil sorption capacity, ranging from 53.2 g/g to 113.8 g/g for various oils and organic solvents. Additionally, the CNF-TA-ICO aerogel demonstrated a significant permeation flux ranging from 123.3 to 473.8 L/m^2^ h. This was followed with separation efficiencies of ranged from 94.4 to 97.1%. These features highlight the enormous potential of the aerogel as an environmentally safe and effective material for the separation of emulsion and oil recovery [135].

**Figure 14:**
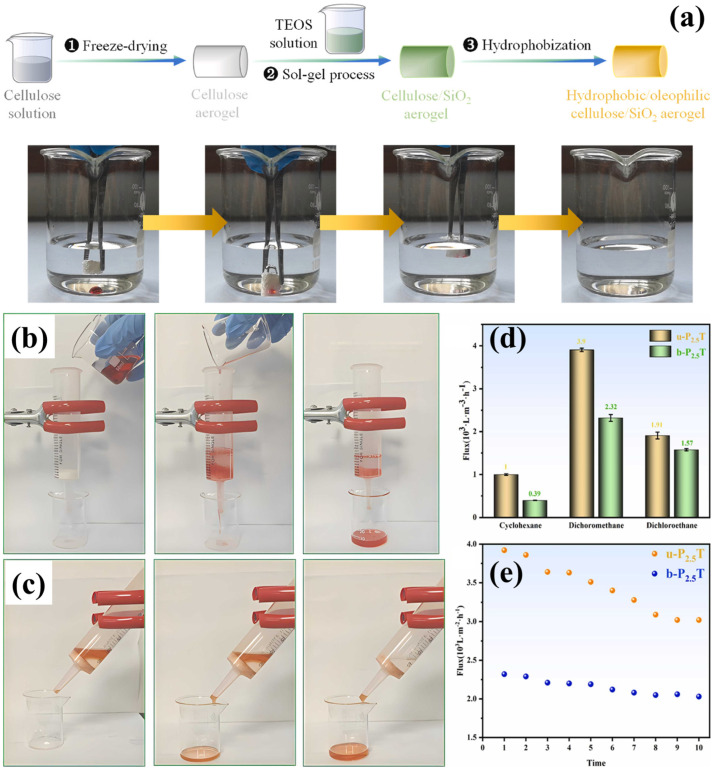
(**a**) Schematic illustration of the preparation process and selective sorption behavior of hydrophobic/oleophilic cellulose−SiO_2_ aerogel. Reprinted with permission from ref. [138]. Copyright © 2024 Elsevier B.V. (License number: 6087010569186). Polyvinyltrimethoxysilane and TEMPO−oxidized cellulose nanofiber aerogel: separation of (**b**) dichloromethane and water and (**c**) n-hexane and water. Flux for (**d**) different oils and (**e**) water and dichloromethane separation for 10 cycles. Reprinted with permission from ref. [139]. Copyright © 2025 The Author(s). Published by Elsevier B.V.

In order to develop hydrophobic antibacterial aerogels based on NFC, Wang et al. [136] developed a dual-functional surface modification approach. The NFC was modified utilizing hexadecyltrimethoxysilane (HDTMS) and cyanuric chloride (CC). CC was used as a precursor for N-halamine functionalities in order to impart antibacterial properties. Additionally, HDTMS was included in the aqueous phase in order to impart hydrophobicity with a low surface energy group. It showed anti-bactericidal action against *Escherichia coli* and *Staphylococcus aureus*. Significantly, over 95% of the antibacterial potency was retained upon rechlorination, despite repeated washing cycles and extensive UV exposure. Furthermore, the aerogels demonstrated extraordinary reusability for the sorption of organic solvents. These were able to maintain a dichloromethane uptake capacity that was higher than 50 g/g even after thirty cycles of absorption and desorption [136]. Zhang et al. fabricated 3D Janus all-cellulose aerogel (3D-JACA) using phase self-assembling (sol–gel process, freeze-drying, and curing) method [137]. In order to create hydrophobic cellulose, the hydrophobic alteration is carried out by combining ethanol, TEOS, and MTMS in a volume ratio of 2:1:1. On one side, 3D-JACA accumulates superhydrophobic (146.8°) and oleophilic properties, whereas on the other side, it accumulates superhydrophilic and oleophilic properties. The 3D-JACA material has been shown to have a high porosity of 97.42% and an extremely low density of 0.041 g/cm^3^. In order to improve oil–water separation utilizing a gravity-driven technique, 3D-JACA high porosity and uneven wettability boost the demulsification effect. This results in a separation effectiveness of 99.51% and a permeance flux of 3111 L/m^2^h. Moreover, a maximum oil sorption capacity of 34.38 g/g is also possessed by the aerogel. Using a gravity-driven technique, it was able to effectively separate a wide variety of O/W and W/O emulsions [137]. In another approach, cellulose-SiO_2_ aerogels have been fabricated through a freeze-drying process [138]. Through the use of vaporized MTMS, the cellulose solution containing layered silica and hydrophobic modification was created as shown in Figure 14a. Furthermore, the porosity dropped from 90.2% to 84.48%, while the aerogel density increased from 28.92 to 34.83 mg/cm^3^ as the cellulose content increased from 0.5 to 1.25 wt%. The surface-engineered aerogels had outstanding wettability qualities, demonstrating an increased WCA of 143° and oil contact angles of 0°. The created aerogels possessed both superhydrophobic and superoleophilic features. In a similar manner, the aerogels demonstrated significant absorption capabilities, which ranged from 16 to 40 g/g for a variety of oils and organic solvents, demonstrating their appropriateness for use in oil spill cleanup and organic pollutant recovery applications (Figure 14a) [138]. Zheng et al. [139] engineered multifunctional aerogels by tailoring TEMPO-oxidized cellulose nanofibers (TOCNF) with polyvinyltrimethoxysilane (PVTMS) using a Pickering emulsion templating method. The PVTMS-TOCNF aerogels exhibited super elasticity and tunable hydrophobicity with a WCA of 130.1°. By adjusting PVTMS-TOCNF ratios and freeze-drying conditions (uni- vs. bidirectional), porous lamellar structures with tunable densities (17–34 mg/cm^3^) were obtained and provided efficient oil–water separation (Figure 14b–e). This aerogel provides a considerable compressive recovery (up to 95%). Notably, flux up to 3900 L/m^2^ h and 99.9% efficiency were obtained for the bidirectional aerogels. As a result of its enhanced surface area, the aerogel exhibited an outstanding oil sorption capacity of between 34 and 67 g/g [139]. In another approach, TOCNF with Ti_3_C_2_T_x_ as a composite aerogel exhibited effective oil–water separation [140]. The composite of cellulose-based aerogels can significantly modify the surface properties of the aerogels, thereby altering the interaction between oil/water and enhancing separation performance. Table 1 represents the summary of the materials, properties, parameters, and findings of oil–water separation performance for cellulose-based aerogels.

## 6. Concluding Remarks and Future Prospects

Aerogels made of cellulose combine the best features of renewable bio-resources with desirable properties such as exceptional porosity, tunable surface chemistry, and low density, making them a promising and environmentally friendly material for efficient oil/water separation. Recent cellulose-based aerogels with unusual wetting characteristics have the potential to change oil/water separation activities. This review highlights the cellulose-based aerogels development, processing, properties, performance, and future prospects. A particular emphasis has been made on the fabrication of cellulose and cellulose-based composite aerogels (in conjunction with other biopolymers, synthetic polymers, inorganic materials, and silane functionalization), oil sorption capabilities, and the separation efficiencies of these materials. Recent investigation indicates that cellulose-based aerogels could potentially be useful in environmental cleanup because of their high absorption efficiency, rapid separation, and great recyclability. Aerogels made of cellulose have the potential to replace current materials and provide distinct advantages in the treatment of oily wastewater, which is a major environmental problem. Nevertheless, there are still obstacles to overcome in order to improve the mechanical robustness, long-term stability, and scalability of aerogels based on cellulose-based materials for use in industrial applications. As a result, conducting more research and making advancements in the field of cellulose-based aerogels is necessary for the future separation of oil and water. In order to generate hydrophobicity, cellulose aerogels are often modified by the addition of silanes, fluorinated compounds, or NPs. On the other hand, certain hydrophobic modifications employ hazardous substances or non-biodegradable components, which may compromise the overall sustainability and environmental compatibility of the material. Therefore, it is essential to identify innovative strategies in surface modifications that prioritize sustainability by using non-toxic agents and/or removing toxic chemicals. Freeze-drying and supercritical drying are two examples of the majority of laboratory-scale manufacturing procedures, which demand a substantial amount of energy and time. This presents challenges for the manufacture of large quantities of the product. In the near future, several production processes, such as ambient drying or simpler chemical crosslinking, need to be established for industrial applications. These approaches will be more cost-effective, scalable, and energy-efficient than traditional methods. Furthermore, the adoption of efficient and scalable production methods like 3D printing, ambient drying, and template self-assembly should be explored to lower costs and enhance uniformity. In order to accommodate a wide range of environmental conditions, including pH, salinity, temperature, and others, it is essential to develop aerogels that might be referred to as “smart” or “sensitive”. There is a need for more study that incorporates unique concepts in order to fully comprehend the potential of hybrid cellulose-based aerogels for sustainable oil–water separation technologies. The determination of the efficient cellulose-based aerogels can be enhanced through the following concepts: In the past, there have been very few cellulose materials/sources that have been thoroughly investigated for their potential application in oil–water separation. In addition to the cellulose materials represented in this review, it is essential to take into consideration the various types of cellulose material based on the intrinsic properties. It is essential to consider the cross-linked structure of aerogels to enhance both separation capabilities and durability. Selecting a cross-linker with a high connecting ability with cationic/anionic/zwitterionic behavior can present a significant advantage. Enhancing the lipophilic-hydrophobic (superhydrophobic-superoleophilic), hydrophilic-lipophobic (superhydrophilic-underwater oleophobic), or other specific wetness forms of the cellulose aerogels through the impact of various surface modifications with different concepts excluding silylation agents. The process of adding functionalized nanomaterials, essential oils, and organic compounds within the aerogels. Functionalized and modified surface properties combined with tunable porosity and a high surface area can potentially significantly change the cellulose-based aerogels oil–water separation. For the purpose of enhancing the performance of cellulose-based aerogels, the discovery of a superior structure with adjustable qualities of metal–organic frameworks (MOF) and covalent organic frameworks (COF) could represent a potential choice. The key challenges associated with cellulose-based aerogels for large-scale application (including raw material variability and standardization, complex and energy-intensive fabrication processes, solvent use and environmental concerns, mechanical stability and durability, surface functionalization and performance trade-offs, scalability of fabrication techniques limitations, cost and economic viability, microbial contamination, lack of industry-standard testing and certification, and regulatory and environmental concerns) must be effectively addressed to enable cellulose-based aerogels most efficient and practical utilization.

## Figures and Tables

**Table 1:** Summary of cellulose-based aerogels properties and oil–water separation performance results.

Materials	Density	Specific Surface Area	Porosity	WCA	Mechanical	Flux	Efficiency	**Sorption**	**Ref.**
CNF/PDA/TiO_2_/OTMS	0.1171 g/mL	−	87.6723%	113.9°	41.83 kPa	23.8095 L/hg	96.15%	59.9 (g/g)	[25]
CNC, PEG, Maleic anhydride, DMAP	31.5 mg/cm^3^	−	96.7%	−	−	−	>97%	−	[38]
Hydroxyethyl cellulose, alkali lignin, epichlorhydrin	0.0198–0.0838 g/cm^3^	−	91.87–98.13%	−	−	7646 ± 167 L/m^2^h	>99%	−	[63]
Natural cellulose fibers, N-methyl morpholine-N-oxide monohydrate (NMMO·H_2_O)	8–10 mg/cm^3^	768.89 m^2^/g	−	151.5°	−	11,718.8 L/m^2^h (carbon tetrachloride (CCl_4_))	−	−	[91]
Kapok fiber/regenerated cellulose (carbon aerogel)	−	−	−	−	−	−	−	98–232.1 (g/g)	[92]
Cellulose (kapok fibers), N-methyl morpholine-N-oxide monohydrate, deep eutectic solvent	3.78 mg/cm^3^	−	98.9%	144.7°	50% strain at 100 cycles	21,972.7 L/m^2^h	−	137.5–371.7 (g/g)	[93]
Bacterial cellulose	5.694 mg/cm^3^	−	98–99%	−	−	−	−	−	[94]
Bacterial cellulose, 1,2,3,4-butanetetracarboxylic acid, MTMS	5.2 mg/cm^3^	63.4 m^2^/g to 49.7 m^2^/g	99.7%	142°	−	−	−	74–165 g/g	[95]
Bacterial cellulose/γ-(2,3-epoxypropoxy) propytrimethoxysilane trimethoxysilane	4.5 and 14.7 mg/cm^3^	6.5 m^2^/g	98.9–99.7%	146.8°	−	−	−	66 mg/g	[97]
Bacterial cellulose	10.25 mg/cm^3^	85.25 m^2^/g	99.4%	0°					[96]
Bacterial cellulose/MTMS	10–15 mg/cm^3^	88.07 m^2^/g	99.1%	>120°		162 L/hg	−	65–156 (g/g)	
sulfonated nano-fibrillated Bacterial cellulose/MTMS	−	6.039 m^2^/g	−	152.4°	−	−	98.48%	42.14–85.37 (g/g)	[98]
BC, Ti_3_C_2_T_x_, MTMS	0.06 g/cm^3^	−	96.5%	136°	−	630 kg/m^2^h^1^	−	−	[99]
Bacterial cellulose/CMC/PDMS	0.097 g/cm^3^	−	96%	145.8°	0.70 MPa at 80% strain	−	−	484 mg/g	[100]
NFC, chitosan	18.6 mg/cm^3^	−	98.7%	−	42.8 kPa	−	>99%	−	[102]
Nanocrystalline cellulose, chitosan,	22.22 kg/m^3^ to 40.82 kg/m^3^	−	≥97.66%	−	−	−	>99.9%	−	[103]
Nanofibrillated cellulose, chitosan,	0.008 g/cm^3^	58.095 m^2^/g	99.46%	−	87.16 kPa at 80% strain	0.826 L/m^2^s^1^	>99.96%	−	[104]
Cellulose nanofibers/chitosan/methyltrimethoxysilane (MTMS), Zinc acetate (Zn (CH_3_COO))_2_	15.87 mg/cm^3^	5.51 m^2^/g	99.01%	132.6°	−	13,167.5 L/m^2^h (CHCl_3_)	98.5%	35–75 g/g	[105]
Wood based cellulose, chitosan	−	−	−	−	−	139,100 L/m^2^h	99.90%	−	[106]
Cellulose nanofibers (CNF)/chitosan/trimethylchlorosilane (PMTS)	−	−	−	141°	−	18,000 to 28,000 L/m^2^h	99.3%	65 (g/g)	[107]
Polyamidoamine (PAMAM)-modified chitosan/cellulose/methyltrimethoxy-silane (MTMS)	0.06–0.11 g/cm^3^	1.93–12.56 m^2^/g	−	139.5°	2.28 MPa	5501.85 L/m^2^h (carbon tetrachloride/water), 4198.60 L/m^2^h, 96.67% (water-in-oil emulsions)	96.67% (water/oil emulsion)	1.49–12.07 (g/g) (CCl_4_)	[108]
Hydroxyethyl cellulose, alkali lignin, epichlorohydrin, n-dodecyl mercaptan (NDM), Fe_3_O_4_/PDA	0.0443 g/cm^3^ to 0.0718 g/cm^3^	−	−	111.18°	−	2986 L/m^2^h	>99%	−	[109]
Cellulose/lignin, Methyltrichlorosilane (MTCS)	15.3 ± 0.7 mg/cm^3^	3.2 m^2^/g	98.87%	168°	11.6% (plastic deformation)	>1000 L/m^2^h	−	38.6–87.9 (g/g)	[110]
Sodium CMC, SA, TiO_2_, CaCl_2_	−	−	−	−	−	7650 L/m^2^h	99.9%	−	[113]
Cellulose nano fibers/Sodium alginate, Methyltrimethoxysilane (MTMS)	24.2 mg/cm^3^	−	97.85%	144.5°	340 kPa at 90% strain	−	−	88.91 (g/g)	[114]
CNC/PVA/TEOS	0.017 g/cm^3^	76 m^2^/g	98.42%	−	−	−	−	69–168 g/g	[118]
Cellulose, PVA, N,N’- methylenebisacrylamide (MBA) and methyltrichlorosilane (MTCS)	50.4 mg/cm^3^	−	96.6%	156.6°	490.7 kPa at 90% strain	7176.3 L/m^2^h	98.5%	−	[119]
CNF/PVA/ethyltrimethoxysilane	10.8 kg/cm^3^	27.9 m^2^/g	98.4%	148°	−	−	−	−	[120]
CNF/PVA/TEMPO/DTOS	11.4 kg/m^3^		99.2%	152°	−	−	−	−	[121]
PVA/cellulose/methyltrichlorosilane	−	−	−	−	−	631.9–2368.7 L/m^2^h	−	−	[122]
Carboxymethyl cellulose/PVA/SiO_2_/Fe^2+^	0.0211 g/cm^3^	132.13 m^2^/g	98.62%	139°	−	19,130 L/m^2^h	−	−	[124]
Nanofibrillated cellulose, 3-(3′-acrylicacidpropylester)-5,5-dimethyl hydantoin (APDMH), poly(ethyleneimine) (PEI), 3-glycidoxypropyltrimethox (GPTMS)	67 mg/cm^3^		94%	−	Recover to 96.76% after 5 compression-release cycles	9500 L/m^2^h	99%	−	[125]
Vinylated nanofibrinogen cellulose/Horseradish peroxidase/modified polyethyleneamine, Vinyltrimethoxysilane (VTMS)	55.1 mg/cm^3^	−	95.5%	0°	42.0 kPa	5000 L/m^2^h	99%	−	[126]
NFC, Polyethyleneimine, methyl trichlorosilane (MTS), Ethylene glycol diglycidyl ether (EGDE)	53.80 mg/cm^3^	−	95.73%	130.0°	Elasticity (95.86%)	5000 L/m^2^h	99%	−	[127]
CNF, GPTMS, PEI, fluorine-contained compound (FS-60)	0.0256 g/cm^3^	127.87 m^2^/g	98.30%	−	−	9060 L/m^2^h	99%	−	[128]
Cellulose nanofibers/TEMPO/PEI/Tannic acid/MTMS	2.96 ± 0.31 kg/m^3^	10–284 m^2^/g	46.45 ± 0.18%	−	−	−	−	102.8 (g/g)	[129]
Cellulose, Dopamine hydrochloride (DA), trimethylchlorosilane (TMCS), Methyltrimethoxysilane (MTMS)	0.0405 g/cm^3^	−	−	142°	1804.5 Pa and 59.0% resilience	3121 L/m^2^h	99.5%	−	[130]
Polyurethane, Fe_3_O_4_, Cellulose	−	−	−	−	−	48,750 L/m^2^h	>97.68%	−	[132]
CNF, SiO_2_,MTMS	6.43 mg/cm^3^	−	99.6%	168.4°	−	1910 ± 60 L/m^2^h	99.5%	−	[133]
CNF, PDMS	22.7 mg/cm^3^	−	−	163.5°	−	2800 L/m^2^h	99.9%	24 to 48 (g/g)	[134]
CNF, tannic acid, silylated castor oil, 3-isocyanatopropyltriethoxysilane (IPTES)	24.0 mg/cm^3^	−	98.32%	135.6° ± 0.8°	−	123.3 to 473.8 L/m^2^ h	94.4% to 97.1%	53.2 to 113.8 g/g	[135]
Cellulose nanofiber/SiO_2_/MTMS	34.83 mg/cm^3^	−	84.48%	143°	−	−	−	−	[138]
CNF/TEMPO/PVTMS	23.5 (b.a) 18.6 (u.a)	16.0 (b.a) 12.6 (u.a)	98.1% (b.a) 98.5 (u.a)	115.6° (b.a) 112.4 (u.a)	−	3900 L/m^2^h (unidirectional aerogel)	−	34–66 (g/g) (bidirectional aerogel)	[139]
NFC, cyanuric chloride, hexadecyltrimethoxysilane (HDTMS)	−	−	−	−	−	−	−	50 g/g	[136]
Janus all-cellulose/methyltrimethoxysilane (MTMS) and tetraethylsilicate (TEOS)	0.041 g/cm^3^	−	97.42%	−	−	3111 L/m^2^h	99.51%	−	[137]
Microfibrillated cellulose/MTMS	6.06–12.21 mg/cm^3^	10.87 m^2^/g	99.33–99.58%	133.15°	27.84 kPa	−	−	66–122 g/g	[141]

## Data Availability

No new data were created or analyzed in this study.

## Abbreviations

The following abbreviations are used in this manuscript:

2D	Two-dimensional
3D	Three-dimensional
ADP	Ammonium dihydrogen phosphate
APDMH	3-(3′-acrylicacidpropylester)-5,5-dimethyl hydantoin
BC	Bacterial cellulose
bPEI	Branched polyethyleneimine
BTCA	1,2,3,4-butanetetracarboxylic acid
CA	Contact angle
CC	Cyanuric chloride
CMC	Carboxymethyl cellulose
CNC	Cellulose nanocrystals
CS	Chitosan
CVD	Chemical vapor deposition
DES	Deep eutectic solvent
DNS	Deep eutectic solvent—N-methyl morpholine-N-oxide monohydrate
DPPA	Dipentaerythritol pentaacrylate
DTOS	Dodecyltrimethoxysilane
ECH	Epichlorhydrin
ETOS	Ethyl trimethoxysilane
GA	Glutaraldehyde
GL	Glycerol
GO	Graphene oxide
GPTMS	3-glycidoxypropyltrimethoxysilane
HBCA	Hydrophobic BC-based aerogel
HDTMS	Hexadecyltrimethoxysilane
ICO	Silylated castor oil
IPTES	3-isocyanatopropyltriethoxysilane
JACA	Janus all -cellulose aerogel
LCMA	Lignin containing cellulose aerogel
MOF	Metal–Organic Framework
MPa	Megapascal
mPEI	modified polyethyleneimine
MTCS	Methyltrichlorosilane
MTMS	Methyltrimethoxylsilane
MWCNT	Multi-Walled Carbon Nanotube
NFC	Nanofibrillated cellulose
NM	Natural microfibrils
NMMO⋅H_2_O	N-methyl morpholine-N-oxide monohydrate
NP	Nanoparticle
OCA	Oil contact angle
OTMS	Octadecyltrichlorosilane
PDA	Polydopamine
PDMS	Polydimethylsiloxane
PDSO	Poly(dodecylsiloxane)
PEG	Polyethylene glycol
PEI	Polyethyleneimine
PLA	Polylactic acid
PMTS	Polymethyltrimethoxysilane
PNC-CS	Polymeric nanocross-linked chitosan
PU	Polyurethane
PUF	Polyurethane foam
PVA	Polyvinyl alcohol
PVTMS	Polyvinyltrimethoxysilane
QHS	Quaternarized N-halamine siloxane
RCA	Regenerated cellulose
SA	Sodium alginate
SBC	Silanized bacterial cellulose
SBCA	Silylated bacterial cellulose aerogels
SHI–OP	Superhydrophilic–oleophobic
SNBC	Sulfonated nanofibrillated bacterial cellulose
SiO_2_	Silica
TA	Tannic acid
TEOS	Tetraethyl orthosilicate
TiO_2_	Titanium dioxide
VNFC	Vinylated nanofibrillated cellulose
WCA	Water contact angle
